# Polarity-Guided Extraction of *Crithmum maritimum* L.: Phenolic Profiling, Selective Anticancer Activity in AR42J and HT-29 Cells, and Antimicrobial Effects

**DOI:** 10.3390/molecules31213877

**Published:** 2026-10-27

**Authors:** İlknur Yılmaz, Fatmanur Yazıcı, Emine Gülçin Köse, Sevde Nur Üçüncü, Sude Büşra Köseoğlu, Funda Bilgili-Tetikoğlu, Yakup Kara, Nevzat Batan, Dimitris Mossialos, Selcen Çelik-Uzuner

**Affiliations:** 1 Epigenetics and Cancer Research Group (EPIKA), Department of Molecular Biology and Genetics, Faculty of Science, Karadeniz Technical University, 61080 Trabzon, Türkiye; 2 Department of Pharmacology, Faculty of Pharmacy, Karadeniz Technical University, 61080 Trabzon, Türkiye; 3 Graduate School of Natural and Applied Sciences, Karadeniz Technical University, 61080 Trabzon, Türkiye; 4 Biomics Research Group, Department of Molecular Biology and Genetics, Faculty of Science, Karadeniz Technical University, 61080 Trabzon, Türkiye; 5 Department of Chemistry, Faculty of Science, Karadeniz Technical University, 61080 Trabzon, Türkiye; 6 Microbial Biotechnology-Molecular Bacteriology-Virology Laboratory, Department of Biochemistry & Biotechnology, University of Thessaly, 41500 Larissa, Greece

**Keywords:** *Crithmum maritimum*, anticancer, cytotoxicity, extract, antimicrobial, phenolic compounds

## Abstract

*Crithmum maritimum* L. (sea fennel) (*C. maritimum*) is a halophyte plant rich in phenolic compounds, flavonoids, essential oils, and other secondary metabolites. Despite its traditional medicinal use, no study has comprehensively compared multiple solvent extracts of *C. maritimum* across diverse cancer and non-cancerous cell lines, leaving its solvent-dependent cytotoxic potential and selectivity largely uncharacterized. Therefore, this study aimed to investigate the biochemical composition of different extracts of *C. maritimum* (obtained using ethanol, ethyl acetate, and distilled water) and the cytotoxic effects of these extracts on pancreatic cancer (AR42J) and colon cancer (HT-29) cell lines, compared to a healthy cell line (ARPE19). Additionally, we determined the extracts’ antimicrobial activity against various bacteria and a fungal species. Ethanol yielded the richest and most diverse phenolic profile in *C. maritimum*, while ethyl acetate extracted moderate levels, and the aqueous extract contained only minor amounts, demonstrating that solvent polarity strongly influences the recovery of bioactive phenolic compounds. Ethanol extracts showed the strongest and most consistent cytotoxicity across normal and cancer cells, while ethyl acetate extracts exhibited high and selective activity on cancer cells. Water extract demonstrated selective cytotoxic effects during prolonged incubations. The extracts displayed solvent-dependent antimicrobial activity, with ethanol and ethyl acetate fractions showing potent inhibition, particularly against *Yersinia pseudotuberculosis*, *Candida albicans*, and *Staphylococcus aureus*. In contrast, the water extract showed no activity. Overall, the findings indicate that *C. maritimum* contains bioactive constituents with selective cytotoxicity against cancer cells and microorganisms, depending on the extraction solvent, supporting its potential as a source of natural anticancer and antimicrobial agents.

## 1. Introduction

Cancer is characterized by the uncontrolled growth of cells resulting from abnormalities in the mechanisms of cell growth and differentiation, and it has become a major global health and socioeconomic problem. Researchers are driven to discover new drug molecules due to various factors, such as the severe side effects of existing drugs and treatments, multiple drug resistance, and low selectivity and specificity. Among cancer types, colorectal cancer is the third most common malignancy worldwide and is the second leading cause of cancer deaths, accounting for 9.3% of all cancer deaths [1,2]. Pancreatic cancer ranks sixth in cancer-related deaths in both genders and accounts for approximately 5% of all cancer deaths.

*C. maritimum* is a perennial halophyte plant belonging to the Apiaceae (parsley) family, commonly known as sea fennel, rock samphire, or sea celery. It typically grows in areas close to the seacoast and has a wide distribution range extending to the eastern Black Sea, Aegean, and Mediterranean coasts [3]. The sea fennel plant is highly branched and can reach 30–60 cm in height. It has thick, tuberous rhizome roots that can extend up to 5 m, fleshy, hairless, triangular leaves, yellowish-white flowers that bloom between June and September, and oval-to-elongated fruits that ripen between November and December [3,4]. It has been used throughout history in both traditional medicine and culinary applications and has been valued for preventing scurvy, particularly due to its high vitamin C content [5]. It has traditionally been used by local communities in salads, soups, and sauces, as well as to prepare medicinal decoctions and infusions. Sea fennel essential oil can also be used as a natural preservative in food products, extending shelf life while enhancing health benefits, aroma, and color [6]. It is known that the aerial parts and young branches of *C. maritimum*, in particular, possess properties that act as a diuretic, regulate digestion, act as a laxative, and expel worms [7]. Sea fennel has also been widely recognized for its anti-helmintic properties since ancient times. Recent studies have shown that various extracts obtained from its above-ground parts possess anti-oxidant, anti-inflammatory, anti-viral, antibacterial, antifungal, and cytotoxic activities [8]. In northern regions, a decoction of sea fennel is used in traditional medicine to support the health of the urogenital system and the liver, while in southern Italy, the same decoction is considered a useful remedy for treating whooping cough and the common cold. Additionally, while leaf tea is traditionally used by residents of Central Italy for its purifying, diuretic, and carminative effects, the fruit infusions are used for their stomach-strengthening, digestive, and carminative properties [9].

*C. maritimum* is a rich source of minerals, particularly calcium (Ca), magnesium (Mg), and potassium (K). Ca and Mg minerals play important roles in cellular metabolism, bone structure, and development [4]. The harvested tender leaves and stems can be consumed raw or boiled, and can also be preserved as pickles in olive oil, vinegar, or brine [4]. A beverage prepared by boiling shoots harvested before fruiting is used for urinary tract and prostate inflammation, as well as abdominal pain; meanwhile, an infusion (herbal tea) made from the leaves is used for digestive system and kidney disorders due to its laxative effect [10]. It is also rich in many bioactive compounds such as polyphenols, carotenoids, ω-3 and ω-6 essential fatty acids, vitamins A and B, minerals, and essential oils [4]. Sea fennel essential oils are known to be rich in antimicrobial, trypanocidal, acaricidal, insecticidal, and nematocidal compounds; consequently, they have been reported as a potential preservative in the food, pharmaceutical, and cosmetic industries [6,11]. Sea fennel seed extracts have been shown to possess antibacterial activity against *Bacillus cereus*, whereas leaf extracts displayed antibacterial activity against *Pseudomonas* strains [6]. Furthermore, it was also reported that *C. maritimum* extracts exhibit antifungal effects.

Although *C. maritimum* has been traditionally used for its antioxidant, antimicrobial, and anti-inflammatory properties, modern biomedical research on its anticancer activity remains limited. Most existing studies have focused on the phytochemical characterization of the plant or its antioxidant potential in non-cancer models [12,13]. Only a few reports have evaluated its cytotoxic effects, and these have largely been restricted to a single solvent extract or to a single cancer cell line, such as breast or liver carcinoma cells [14]. The ethyl acetate extract of this plant has been shown to modulate metabolic markers, including AMP-activated protein kinase (AMPK), Sirtuin 1 (SIRT1), and Sirtuin 3 (SIRT3), in hepatocellular carcinoma cells [15]. The application of *C. maritimum* extract in combination with sorafenib (a chemotherapy drug commonly used in liver cancer treatment) in liver cancer cells reduces cell proliferation [16]. Importantly, no comprehensive study has compared multiple solvent extractions (e.g., ethyl acetate, ethanol, and water) across different cancer cell lines of distinct origins and non-cancerous epithelial cells to assess both therapeutic potential and selectivity. Because extract composition varies depending on the solvent used, their cytotoxic effects may differ accordingly. Furthermore, pancreatic AR42J and retinal ARPE19 cells have not previously been utilized to evaluate *C. maritimum* cytotoxicity. Therefore, the purpose of this study is to investigate the cytotoxic effects of extracts prepared from the *C. maritimum* plant using ethyl acetate, ethanol, and water on AR42J (pancreatic cancer cells), HT-29 (colon cancer cells), and ARPE19 (retinal epithelial cells).

## 2. Results

### 2.1. Phenolic Compound Results of C. maritimum Plant Extract

In the present study, several phenolic acids and flavonoids were identified and quantified in the *C. maritimum* L. extracts [17]. Among the hydroxybenzoic acids, protocatechuic acid was the predominant compound, occurring at the highest concentration in the ethanolic extract (267.30 µg/g dry extract), followed by p-hydroxybenzoic acid and gallic acid. Within the hydroxycinnamic acid group, ferulic acid (196.26 µg/g) and p-coumaric acid (91.48 µg/g) were likewise detected in substantial amounts. In contrast, the aqueous extract contained only minor quantities of phenolic acids, while the ethyl acetate extract yielded moderate levels, underscoring the influence of solvent polarity on phenolic extraction efficiency. Among the identified flavonoids, pinocembrin and apigenin were detected in all extracts, again with their peak concentrations found in the ethanol fraction. The predominance of these flavonoids, together with hydroxycinnamic acids, suggests that ethanol effectively recovers both medium- and low-polarity phenolics, contributing to the diversity and richness of the extract. Overall, the ethanol extract exhibited the most complex and abundant phenolic profile, both qualitatively and quantitatively (Table 1). These results indicate that the extraction solvent plays a key role in determining the phenolic composition of *C. maritimum*, demonstrating that ethanol offers an optimal balance between polarity and solubility for the recovery of bioactive phenolic compounds. Detailed compound profiles are provided in Supplementary Table S1.

**Figure 1:**
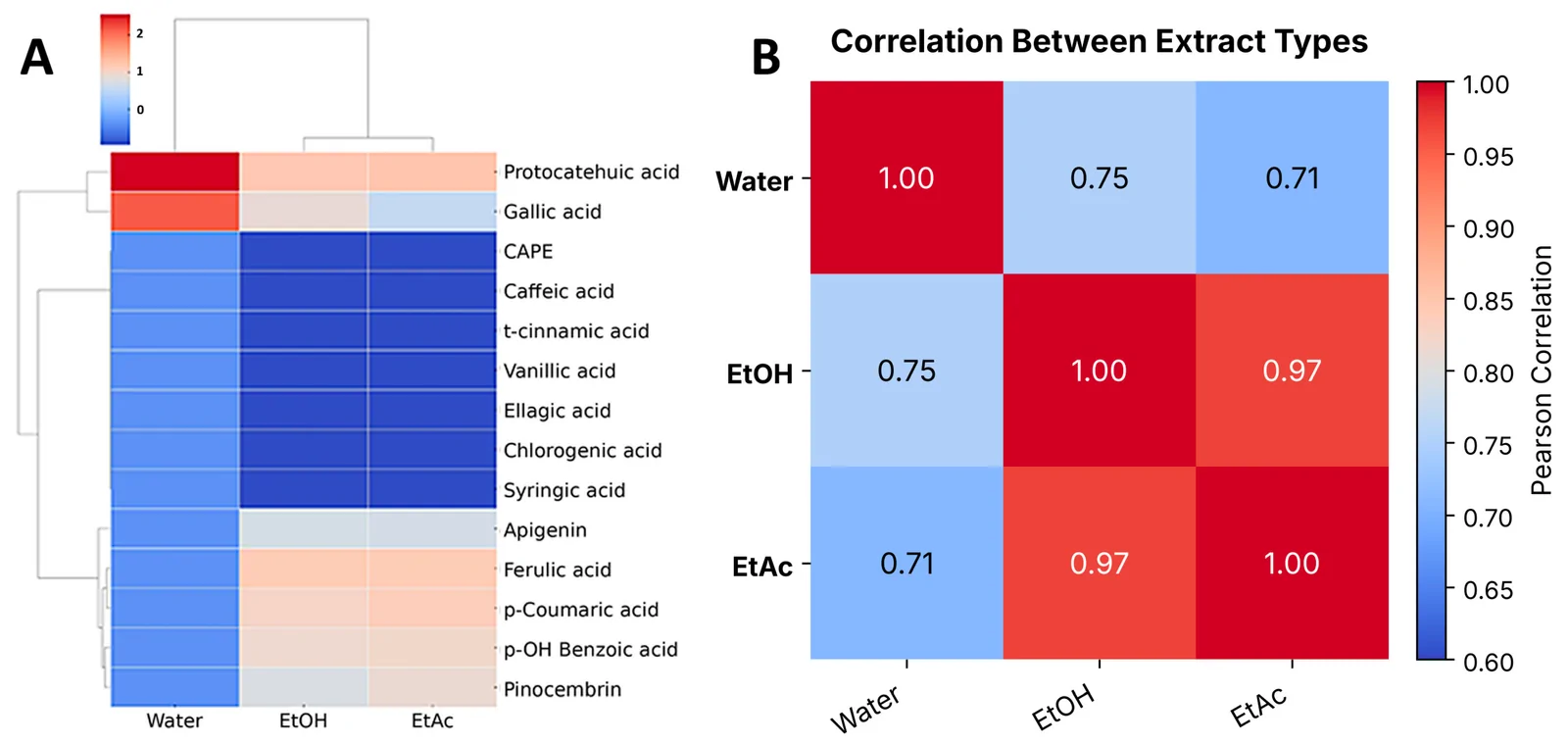
Clustered heat map (**A**) and correlation heat map (**B**). (**A**) and (**B**) show the solvent-dependent distribution of phenolic compounds, and phytochemical similarity across different extracts (W; water, EtOH; ethanol, EtAc; ethyl acetate), respectively.

Based on the HPLC quantification results, a clustered heatmap was generated to illustrate the solvent-dependent distribution of phenolic compounds across aqueous (dH_2_O), ethanol (EtOH), and ethyl acetate (EtAc) extracts. Two major phytochemical clusters were identified, with the EtOH and EtAc extracts showing high similarity due to elevated levels of protocatechuic acid, gallic acid, ferulic acid, p-coumaric acid, and pinocembrin. In contrast, the water extract formed a distinct cluster driven by its comparatively higher relative proportion of protocathechuic acid content. Overall, this pattern indicates that mid-polarity solvents (EtOH and EtAc) exhibit superior extraction efficiency for most phenolic constituents (Figure 1A). The correlation heatmap compares the overall phytochemical similarity among the three solvent extracts. Ethanol and ethyl acetate extracts displayed a strong positive correlation (r = 0.97), reflecting their comparable extraction profiles, whereas water showed lower correlations with both EtOH (r = 0.75) and EtAc (r = 0.71). These results further support the conclusion that EtOH and EtAc share a similar phenolic composition, while the water extract represents a chemically distinct profile (Figure 1B).

Hierarchical clustering analysis of the identified phenolic compounds revealed the presence of three distinct clusters based on their solvent-dependent distribution profiles (Figure 1A). The first cluster was mainly represented by protocatechuic acid and gallic acid, both of which exhibited relatively high abundance across all extract types, with particularly pronounced levels in the aqueous and ethanol extracts. The second cluster comprised caffeic acid phenethyl ester (CAPE), caffeic acid, t-cinnamic acid, vanillic acid, ellagic acid, chlorogenic acid, and syringic acid, which displayed comparatively lower concentrations and closely related distribution trends among the tested solvents. In contrast, the third cluster included apigenin, ferulic acid, p-coumaric acid, p-OH benzoic acid, and pinocembrin, compounds that were predominantly enriched in the ethanol and ethyl acetate fractions. Overall, the clustering pattern clearly demonstrates that solvent polarity plays a critical role in determining both the extraction efficiency and compositional distribution of phenolic constituents. In particular, ethanol and ethyl acetate exhibited similar extraction behavior for several moderately polar compounds, whereas water preferentially enriched highly polar phenolic acids.

### 2.2. Anticancer Activity of C. maritimum Plant Extracts on Human Cells

**Figure 2:**
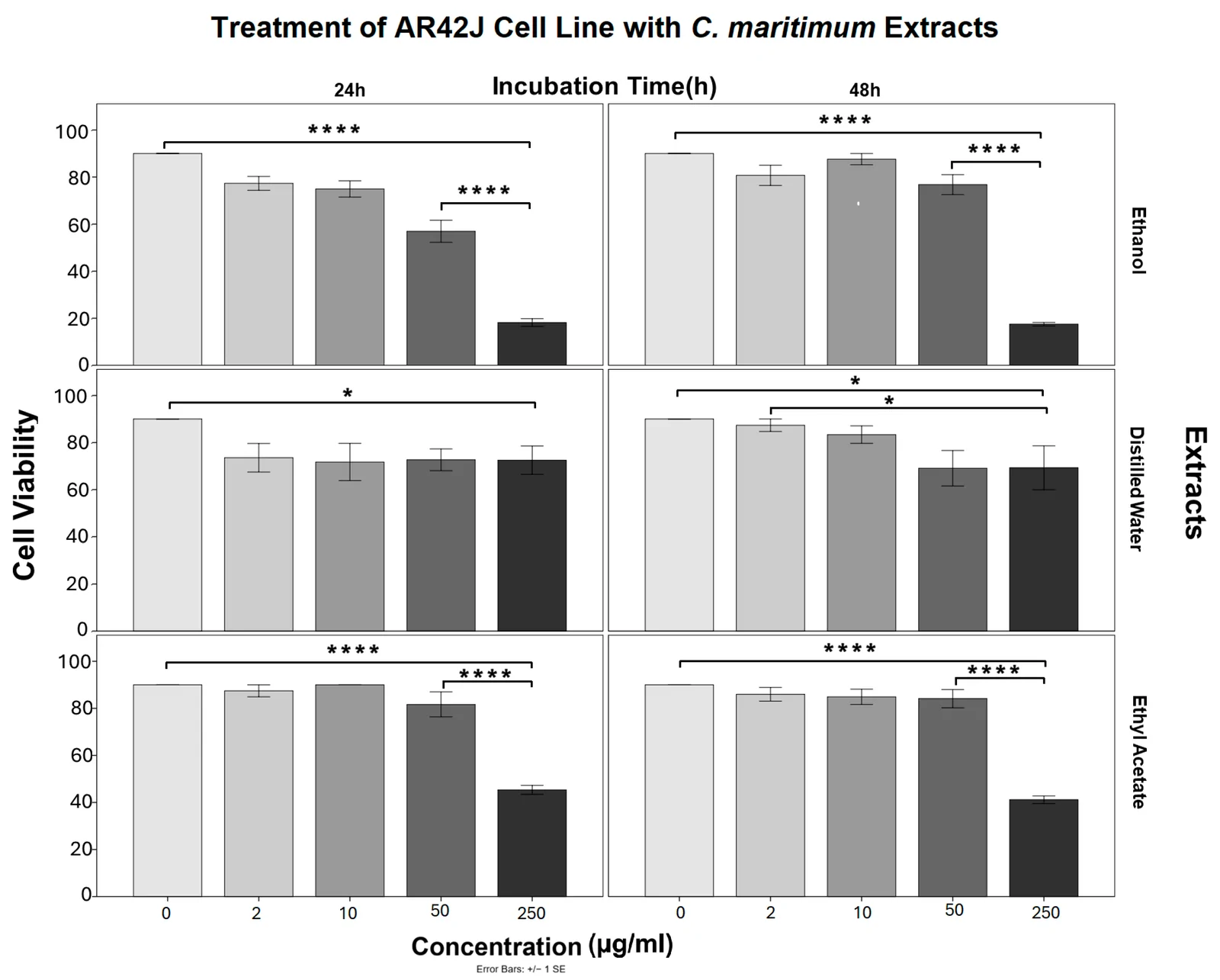
Treatment of AR42J Cell Line with *C. maritimum* Extracts. The cytotoxic effects of three different extracts (ethanolic, aqueous, and ethyl acetate extracts) obtained from the *C. maritimum* (sea fennel) plant on the AR42-J cell line were evaluated at 24- and 48-h incubation times. Cell viability decreased significantly in each extract group as the applied concentration (0–250 µg/mL) increased. Cell viability percentages were arcsine-transformed. Note: The maximum final concentration of ethyl acetate solvent in the 250 µg/mL extract treatment was 0.03% (*v*/*v*). Solvent controls tested at equivalent and higher concentrations (0.03–0.25% *v*/*v*) demonstrated negligible cytotoxicity. *, *p* ≤ 0.05 and ****, *p* ≤ 0.0001.

The cytotoxic effects of the ethanol, aqueous, and ethyl acetate extracts obtained from the *C. maritimum* plant at different concentrations (0–250 µg/mL) on HT-29, ARPE19, and AR42-J cell lines were evaluated following 24 h and 48 h of treatment (cell viability percentages after solvent treatments are given in the Supplementary Figure S1). In the pancreatic cancer cell line AR42J, treatment with the ethanol extract induced the most significant decrease in cell viability at both 24 h and 48 h at concentrations of 50 µg/mL (*p* < 0.0001 and *p* < 0.01, respectively), and at 250 µg/mL (*p* < 0.0001). At the 250 µg/mL concentration, more than a two-fold reduction in cell viability was observed compared to control cells (Figure 2). Higher concentrations caused a more pronounced decrease in viability than lower concentrations; specifically, significant differences were found between 250 µg/mL and all lower concentrations (*p* < 0.0001) at both 24 h and 48 h (Supplementary Figure S2). The aqueous extract also induced a significant decrease in cell viability at 24 h (*p* < 0.05 for all tested concentrations 2 µg/mL, 10 µg/mL, 50 µg/mL, and 250 µg/mL compared to untreated controls) (Figure 2 and Figure S2). After 48 h of treatment with the water extract, only the 50 µg/mL and 250 µg/mL concentrations resulted in a significant decrease compared to untreated control cells (*p* < 0.05). However, no significant differences in viability were observed between different concentrations (*p* > 0.05) after 24 h or 48 h, except between 2 µg/mL and 250 µg/mL (*p <* 0.05) at 48 h (Supplementary Figure S2). This finding suggests that the distilled water extract exhibits lower cytotoxicity in the AR42-J cell line compared to the ethanol extract. On the other hand, cell viability decreased significantly (*p* < 0.05) at 24 h and 48 h after treatment with the ethyl acetate extract, particularly at higher concentrations (50 µg/mL and 250 µg/mL compared to the untreated control). Cell viability at 250 µg/mL was significantly lower than at all lower concentrations (*p* < 0.0001) (Figure 2 and Figure S2). Overall, a consistent pattern of cytotoxicity was observed for AR42J cells at both 24 h and 48 h following treatment with the different extracts, with no substantial difference in the extent of cytotoxicity between the two time points.

**Figure 3:**
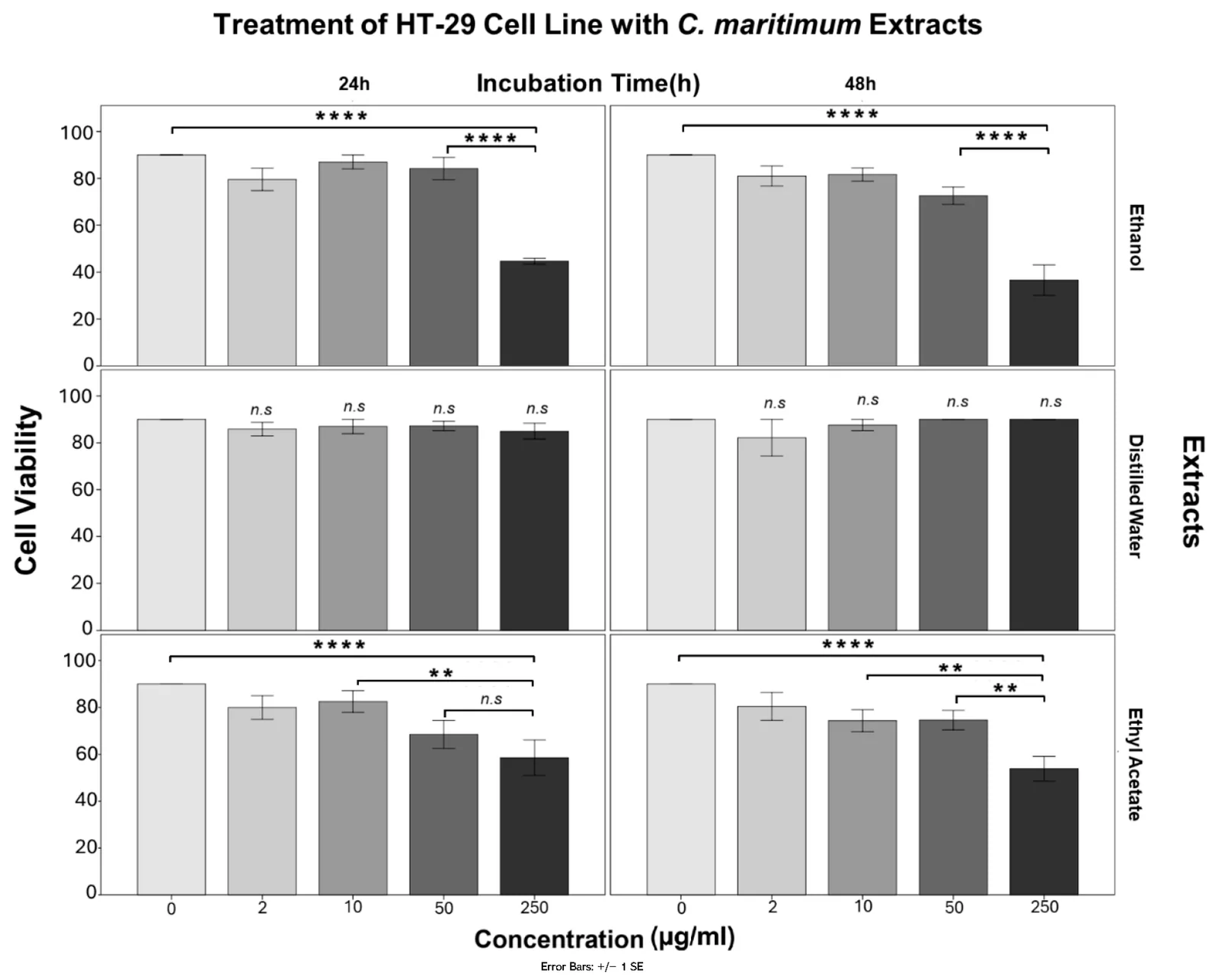
Treatment of the HT-29 cell line with *C. maritimum* plant extract. The cytotoxic effects of ethanolic, aqueous (distilled water), and ethyl acetate extracts obtained from the *C. maritimum* plant on the human colon adenocarcinoma cell line (HT-29) were evaluated at 24- and 48-h incubation periods. (n.s, not significant). Cell viability percentages were arcsine-transformed. Note: The maximum final concentration of ethyl acetate solvent in the 250 µg/mL extract treatment was 0.03% (*v*/*v*). Solvent controls tested at equivalent and higher concentrations (0.03–0.25% *v*/*v*) demonstrated negligible cytotoxicity. **, *p* ≤ 0.01 and ****, *p* ≤ 0.0001.

In the HT-29 colon cancer cell line, treatment with the ethanol extract for 24 h resulted in a significant decrease in cell viability only at the highest concentration (250 µg/mL) compared to untreated control cells (*p* < 0.0001) and all lower concentrations (*p* < 0.0001) (Figure 3). However, upon prolonged incubation (48 h), the 50 µg/mL concentration also induced a significant reduction in cell viability (*p* < 0.01) compared to untreated cells. Cell viability did not change significantly following the treatment with the aqueous extracts (*p* > 0.05), regardless of the concentration and incubation time (Figure 3 and Figure S2). In contrast, the highest two concentrations (50 µg/mL and 250 µg/mL) of the ethyl acetate extract caused significant cell death in HT-29 cells (*p* < 0.01 and *p* < 0.0001, respectively) compared to untreated controls at both 24 h and 48 h. While there was no significant difference between the 50 µg/mL and 250 µg/mL concentrations in inducing cell death at 24 h (*p* > 0.05), at 48 h, the 250 µg/mL concentration caused significantly greater cytotoxicity than 10 µg/mL and 50 µg/mL (*p* < 0.01), and to 2 µg/mL (*p* < 0.0001) (Figure 3 and Figure S2).

**Figure 4:**
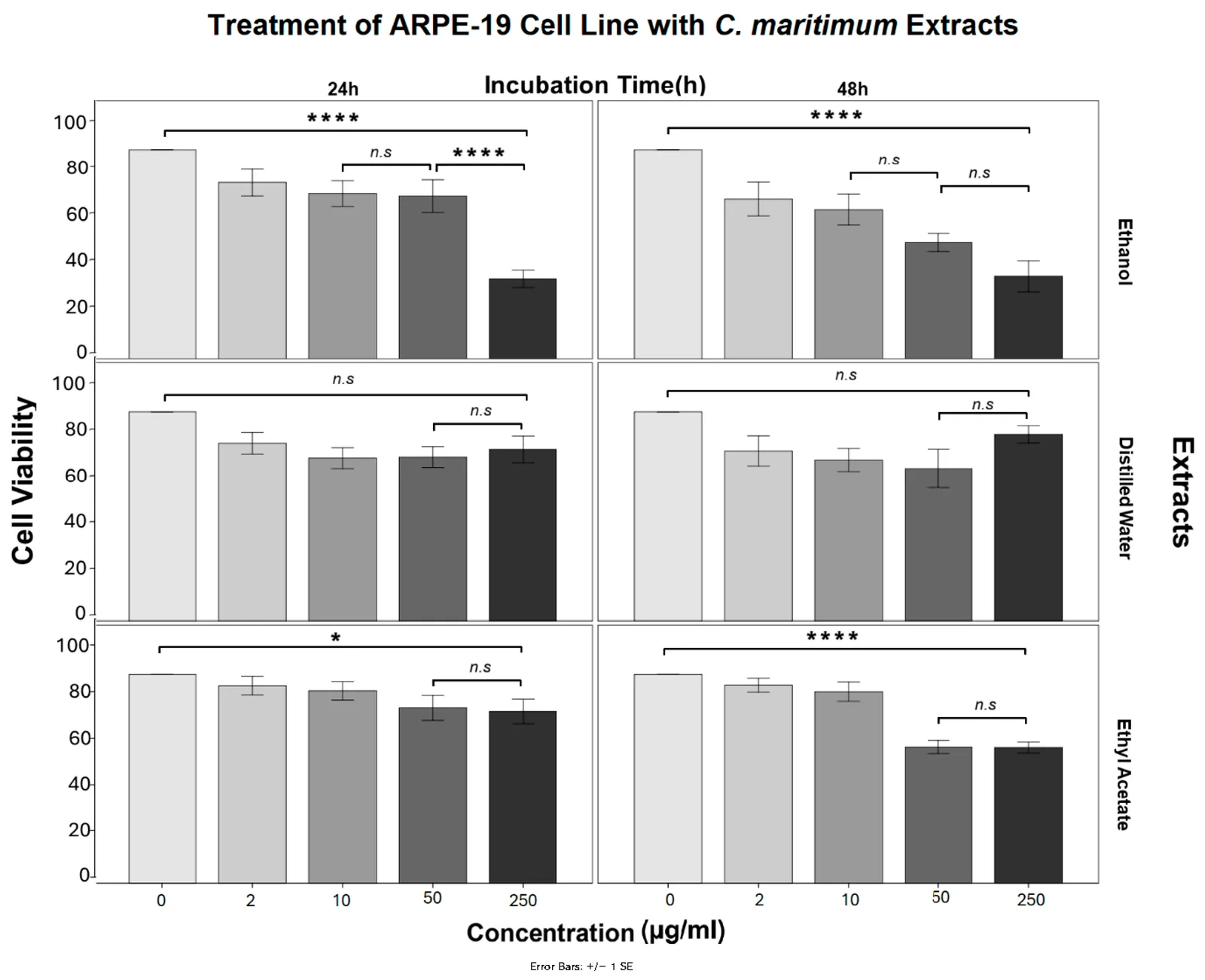
Treatment of the ARPE19 cell line with *C. maritimum* plant extract. The cytotoxic effects of ethanolic, aqueous, and ethyl acetate extracts obtained from the *C. maritimum* plant on the human retinal pigment epithelial cell line (ARPE19) were evaluated at 24- and 48-h incubation times. Cell viability results showed significant differences depending on the applied concentration and extract type. (n.s, not significant). Cell viability percentages were arcsine-transformed. Note: The maximum final concentration of ethyl acetate solvent in the 250 µg/mL extract treatment was 0.03% (*v*/*v*). Solvent controls tested at equivalent and higher concentrations (0.03–0.25% *v*/*v*) demonstrated negligible cytotoxicity. *, *p* ≤ 0.05 and ****, *p* ≤ 0.0001.

We also evaluated the response of non-cancerous human cells, specifically the ARPE19 epithelial cells, to the extracts. In ethanol extract treatments, cell viability remained close to that of the control group at the lowest concentration (2 µg/mL) at 24 h (Figure 4). A significant decrease in viability was observed with increasing concentrations, particularly at 250 µg/mL (*p* < 0.0001). Incubation for 48 h showed a similar pattern; however, the lowest concentration also resulted in a significant reduction in cell death (*p* < 0.05) (Figure 4 and Figure S2). At 24 h, 250 µg/mL induced significantly higher cell death than all other concentrations, and a similar pattern was observed at 48 h, except when compared to 50 µg/mL (*p* > 0.05) (Figure 4). The aqueous extract induced a slight but significant decrease in cell viability at all tested concentrations (*p* < 0.05) at 24 h compared to untreated cells; nonetheless, there were no significant differences among the various concentrations. After 48 h, the cellular response to the aqueous extract was similar across the concentrations; interestingly, viability at the highest concentration did not differ significantly from the untreated control (Figure 4 and Figure S2). Ethyl acetate extracts at the highest two concentrations induced significant cytotoxicity at both incubation time points (*p* < 0.05 for both 50 µg/mL and 250 µg/mL), with a more pronounced reduction in viability after 48 h compared to the untreated control (*p* < 0.0001 for both concentrations). Significant differences among the concentrations were only detected after 48 h, except between 50 µg/mL and 250 µg/mL. All post-hoc test results are presented in Supplementary Figure S2.

**Figure 5:**
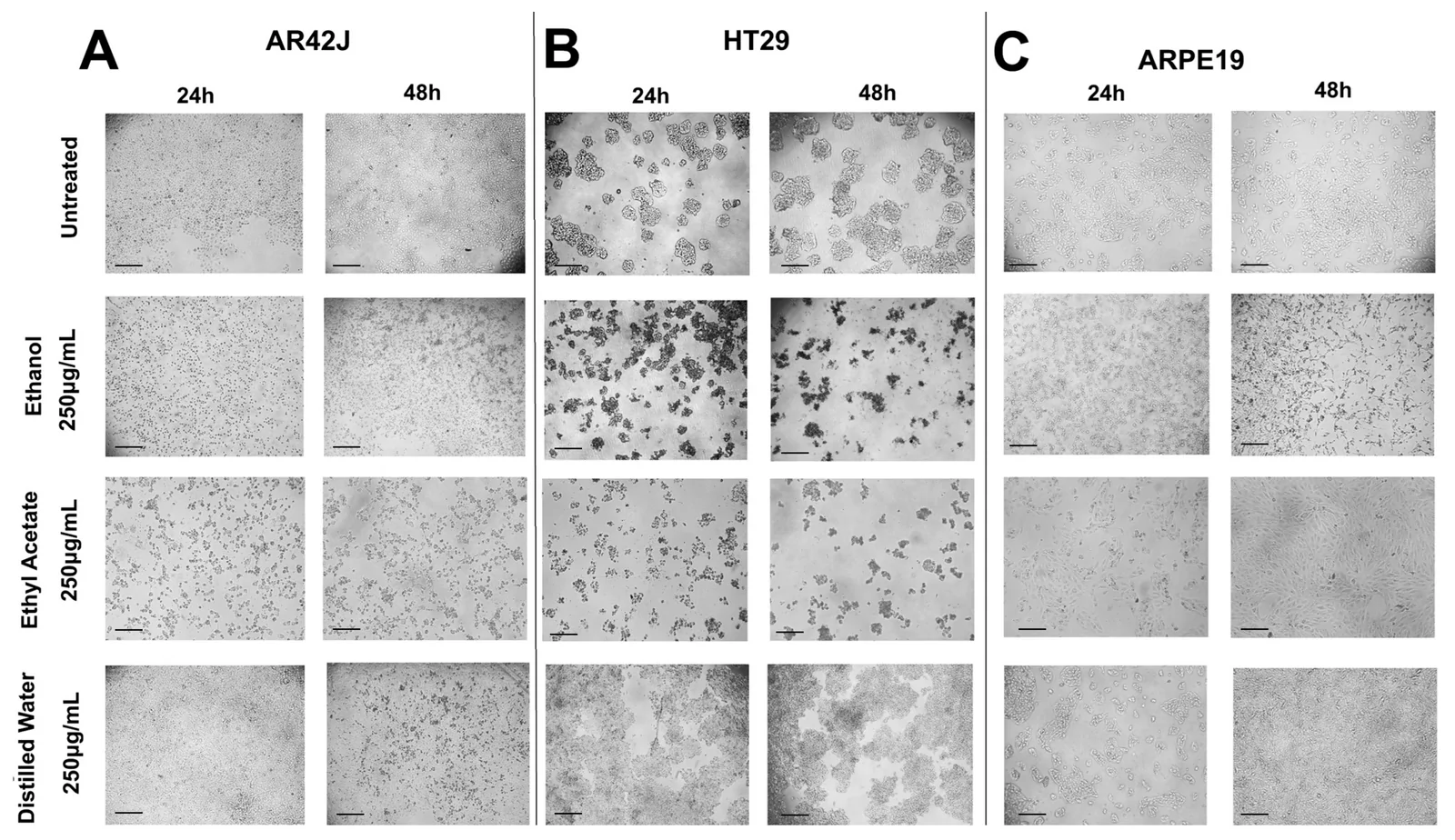
Representative microscope images of (**A**) AR42J (pancreatic cancer), (**B**) HT-29 (colon cancer), and (**C**) ARPE19 (normal epithelial) cell lines after 24 and 48 h of incubation with *C. maritimum* extracts. Scale bar: 100 µm.

Table 2 summarizes IC50 values of cells after the treatments with ethanol, distilled water, and ethyl acetate extracts of *C. maritimum.* The ethanol extract exhibits the strongest and most consistent cytotoxic activity: it achieves moderate IC50 values in all three cell lines at 24 h and generally becomes more potent by 48 h, particularly in HT-29 cells (where IC50 decreased from 88 µg/mL to 72.1 µg/mL). In contrast, the distilled water extract shows very weak cytotoxicity. IC50 values remain above the highest tested concentration (>250 µg/mL) in ARPE19 and HT-29 at both time points, while AR42J cells at 24 h only, indicating minimal cytotoxic potential. The ethyl acetate extract demonstrates intermediate potency, showing no measurable IC50 in ARPE19 and HT-29 cells (>250 µg/mL), but displaying cytotoxic activity in AR42J cells at both time points, with slightly improved potency at 48 h. Overall, the ethyl acetate extract shows selective activity on pancreatic cancer. Figure 5 shows representative microscopy images of each cell line following treatment with the respective extracts.

The selective index (SI) values were also calculated to evaluate the specific cytotoxicity of the extracts at 24 h and 48 h for cancer cells (Table 3). The ethyl acetate extract showed the highest selectivity against pancreatic cancer cells (AR42J) at both 24 h and 48 h, while the water extract showed selectivity only after prolonged incubation (48 h). The ethanolic extract showed cytotoxic effects on both normal and cancer cells, leading to limited selectivity, with slightly higher selectivity observed against pancreatic cancer. These findings suggest that *C. maritimum* is a promising plant, specifically against pancreatic cancer.

### 2.3. Antimicrobial Activity of the Extracts

The antimicrobial activities of *C. maritimum* extracts obtained using different solvents were evaluated against four Gram-negative bacterial strains (*Escherichia coli*, *Yersinia pseudotuberculosis*, *Klebsiella pneumoniae* and *Pseudomonas aeruginosa*), three Gram-positive bacterial strains (*Staphylococcus aureus*, *Staphylococcus epidermidis* and *Bacillus subtilis*), and one yeast-like fungal strain (*Candida albicans*). The antimicrobial profiles and MIC values of the extracts are shown in Table 4. Some extracts did not exert antimicrobial activity against the tested microorganisms; in particular, the aqueous extract displayed no inhibitory effect across all tested strains. On the other hand, the strongest antimicrobial activity of *C. maritimum* was observed against *Y. pseudotuberculosis* using the ethyl acetate extract, with an MIC of 0.01 mg/mL. Furthermore, the MIC against *C. albicans* was determined to be 0.01 mg/mL for both the ethanol and ethyl acetate extracts. The MIC value of the ethanol extract against *B. subtilis* was lower (12.50 mg/mL) than that of the ethyl acetate extract (>31.25 mg/mL). The MIC value of the ethyl acetate extract against *P. aeruginosa* was >31.25 mg/mL, while no MIC could be determined for the ethanol and aqueous extracts. The lowest MIC value (6.125) against *S. aureus* was recorded for both ethanol and ethyl acetate extracts (Table 4). Representative microtiter plates from the INC colorimetric assay are shown in Supplementary Figure S3, and the baseline antimicrobialeffects of the vehicle solvents are presented in Supplementary Figure S4. The highest concentration of the vehicle solvents used did not exert any antimicrobial activity (Figure S4 and Table S2).

## 3. Discussion

This study investigated the cytotoxic and antimicrobial effects of extracts of *C. maritimum* obtained using different solvents, in conjunction with the phytochemical composition of the extracts. *C. maritimum* is a halophyte plant rich in phenolic compounds, flavonoids, essential oils, and other secondary metabolites. Compounds such as chlorogenic acid, neochlorogenic acid, ferulic acid, cryptochlorogenic acid, rutin, quercetin, and limonene have been highlighted as key components of the phytochemical profile of this species. *C. maritimum* is also a rich source of minerals, particularly calcium (Ca), magnesium (Mg), and potassium (K), which play vital roles in cellular metabolism, bone structure, and development [4]. Most of these compounds have been previously isolated from specific plant tissues, such as leaves, flowers, and fruits, typically using hydroethanolic extraction [3]. Souid et al. further reported the presence of quercetin and rutin in their extracts, whereas these compounds were not detected in any of the extracts analyzed in the present study. While their work evaluated exclusively the leaves of plants collected in France, the extracts in our study were prepared from the whole aerial parts collected in Turkey in August 2024. Under our experimental conditions, protocatechuic acid, ferulic acid, p-coumaric acid, and p-hydroxybenzoic acid were identified as the predominant constituents, particularly in the ethanol extract, and to a lesser extent in the ethyl acetate extract. A previous study compared the nutritional value of *C. maritimum* L. across different natural habitats (cliffs, sandy and stony shores) and controlled cultivation conditions in Spain. Despite environmental variations, plants growing in wild habitats maintained a balanced nutritional profile comprising proteins, lipids, and sodium, coupled with a high phenolic content. In contrast, plants grown under controlled conditions demonstrated increased protein and lipid accumulation, but reduced sodium levels and a significant decline in total phenolic compounds [18]. Collectively, these findings suggest that the phytochemical composition of plant extracts can fluctuate depending on geographical, climatic, and seasonal factors, as well as the specific organ or tissue harvested.

The selective cytotoxicity exhibited by the extracts tested in our study, particularly the ethanolic and ethyl acetate extracts, against colorectal and pancreatic cancer cells can be attributed not only to the general interplay between hydrophilic and lipophilic constituents but also, to a large extent, to the potent anticancer properties of the specific phenolic acids and flavonoids identified in these extracts. Among the identified flavonoids, apigenin has drawn considerable attention due to its well-documented ability to modulate multiple cancer-related signaling pathways. Jang et al. demonstrated that apigenin triggers programmed cell death in various cancer types by activating caspases and elevating intracellular reactive oxygen species (ROS) levels [19]. Ahmadzadeh et al. reported that apigenin significantly reduces tumor burden in colorectal adenocarcinoma (CRC) models, decreases in vitro cell viability, and induces cell cycle arrest, particularly in the G2/M and S phases [20]. Similarly, Amini et al. summarized the ability of apigenin to synergistically trigger multiple cell death pathways, including apoptosis, autophagy, senescence, and ferroptosis, in cancer cells [21]. Furthermore, Ghanbari-Movahed et al. investigated the effects of apigenin and related flavonoids (such as isovitexin) on oncogenic metabolism in cancer stem cells (CSCs), demonstrating their capacity to suppress tumor progression and overcome chemoresistance, thereby underscoring their therapeutic potential in oncology [22].

The cytotoxic and antimicrobial activities observed in the ethanolic and ethyl acetate extracts of *C. maritimum* likely reflect the multi-targeted and synergistic actions of their predominant phenolic constituents. Flavonoids such as apigenin [20,21] and pinocembrin [23,24] promote cell cycle arrest and apoptosis by modulating oncogenic survival networks, including the PI3K/Akt/mTOR and NF-kappaB signaling pathways. Concurrently, hydroxybenzoic acids—such as gallic acid [25,26,27], protocatechuic acid [28], 4-hydroxybenzoic acid, and vanillic acid [29,30], disrupt cellular energy homeostasis and promote cell death by triggering ROS generation, inducing mitochondrial membrane dysfunction, activating intrinsic and extrinsic apoptotic cascades, and inhibiting P-glycoprotein-mediated efflux mechanisms. Furthermore, ferulic acid [31,32,33] and p-coumaric acid [34] inhibit cell cycle progression, suppress tumor angiogenesis, induce autophagy [35], and target energy metabolism by downregulating aerobic glycolysis via the lncRNA495810/PKM2 axis [36]. Additionally, protocatechuic acid and quercetin exhibit anti-inflammatory, anti-metastatic, and anti-pyroptotic activities within the tumor microenvironment [37]. Collectively, solvent-dependent variations in the recovery of these bioactive metabolites explain the enhanced potency of organic extracts compared to aqueous fractions.

In the present study, the selective cytotoxicity exhibited by *C. maritimum* extracts, particularly the ethanol and ethyl acetate fractions, against cancer cell lines (HT-29 and AR42J) may not be explained solely by the general interplay between hydrophilic and lipophilic constituents. Instead, the observed activity aligns with reports that specific phenolic acids and flavonoids identified in our extracts may contribute to these biological effects. For instance, the potential contribution of identified flavonoids, such as apigenin and pinocembrin, is supported by literature indicating that these compounds can promote promote cell cycle arrest and apoptosis in cancer cells through suppression of oncogenic survival pathways, including PI3K/Akt/mTOR and NF-κB signaling [20,21,23,24]. Likewise, compounds such as gallic acid and protocatechuic acid have been reported to induce mitochondrial dysfunction, elevate intracellular ROS production, activate intrinsic apoptotic cascades, inhibit cell proliferation, and suppress inflammatory forms of cell death, including pyroptosis and necroptosis [26,28,37]. Furthermore, our findings are consistent with reports suggesting that ferulic acid and p-coumaric acid may disrupt tumor energy metabolism by inhibiting aerobic glycolysis through the PKM2-associated pathway [36], induce cell cycle arrest [33], and simultaneously promote both autophagy and apoptosis [35]. Collectively, the pronounced cytotoxic and antimicrobial activities observed in the ethanol and ethyl acetate extracts, compared with the weaker activity of the aqueous extract, may be attributed to solvent-dependent differences in the recovery of these major secondary metabolites. Although the potential cellular mechanisms of the identified key constituents were contextualized and discussed in light of current literature, direct testing of these individual pure compounds could be explored in future studies. Therefore, although single individual compounds were not tested separately in this study, the high bioactivity observed may hypothetically reflect the multi-targeted and potentially synergistic actions of the identified phenolic constituents against both microbial pathogens and tumor cells.

**Figure 6:**
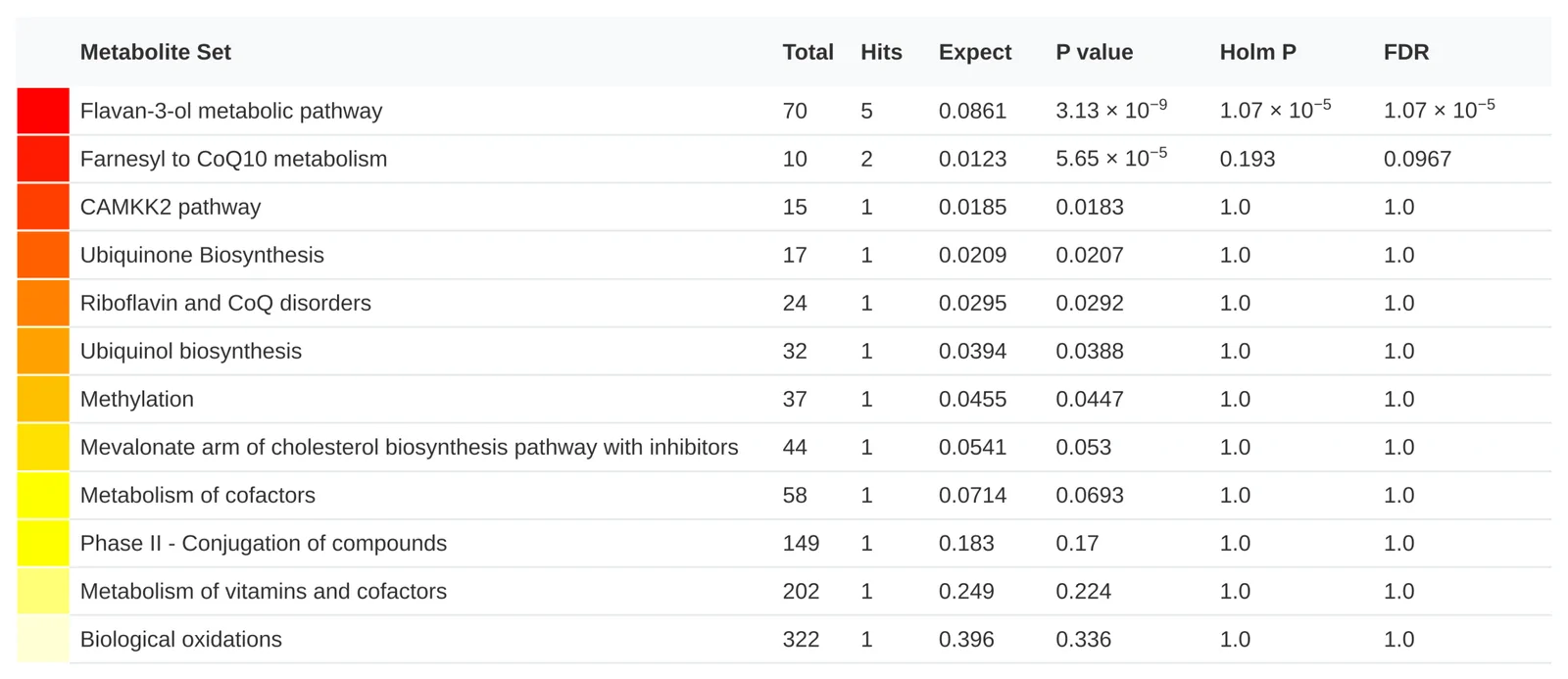
Enrichment analysis of p-OH benzoic acid, protocatechuic acid, gallic acid, ferulic acid, p-coumaric acid, apigenin, and pinocembrin by MetaboAnalyst 6.0.

To further elucidate the potential biological significance of the identified secondary metabolites, a pathway enrichment analysis was conducted using the RaMP-DB database within MetaboAnalyst 6.0 (https://www.metaboanalyst.ca/, accessed on 21 October 2026), focusing on the predominant phenolic compounds present in the ethanol and ethyl acetate extracts (namely p-hydroxybenzoic acid, protocatechuic acid, gallic acid, ferulic acid, p-coumaric acid, apigenin, and pinocembrin). Among these, five metabolites (gallic acid, 4-hydroxybenzoic acid, 4-hydroxycinnamic acid, ferulic acid, and protocatechuic acid) were significantly enriched in the flavan-3-ol metabolic pathway (*p* < 0.0001), while two metabolites (4-hydroxycinnamic acid and 4-hydroxybenzoic acid) were involved in the farnesyl to CoQ10 metabolic pathway (*p* < 0.0001) (Figure 6). The enrichment of these pathways provides mechanistic insights into the biological activities observed in the present study. Flavan-3-ol metabolism is intrinsically linked to the generation of bioactive phenolic metabolites that possess potent antioxidant, anti-inflammatory, and reported anticancer properties. These metabolites have been shown to modulate oncogenic signaling pathways, cellular redox homeostasis, and gene expression patterns regulating tumor initiation and progression [38,39]. In addition, the farnesyl-to-CoQ10 pathway is critical for the biosynthesis of coenzyme Q10, a key electron transporter in the mitochondrial electron transport chain and an important lipid-soluble antioxidant. Given that mitochondrial bioenergetics and redox regulation play central roles in modulating cancer cell survival and apoptosis, the enrichment of this pathway further reinforces the potential contribution of these identified phenolic compounds to the cytotoxic effects exerted by the ethanol and ethyl acetate extracts [40].

The findings of the present study demonstrate that the cytotoxic activity of *C. maritimum* extracts is strongly influenced by the solvent polarity, extract concentration, and incubation period, underscoring the importance of extraction polarity in determining biological activity. Vehicle solvent controls were evaluated at the same final concentrations as those present in the extract treatments (Figure S1). Specifically, ethyl acetate showed moderate intrinsic cytotoxicity at the highest concentration tested (0.25% *v*/*v*), reducing HT-29 cell viability to approximately 63–68%. Therefore, the decrease in viability observed with the 250 µg/mL ethyl acetate extract may be partially influenced by solvent-related effects and should be interpreted accordingly. Among the tested extracts, the ethanol extract showed the strongest cytotoxic effects against both AR42J and HT-29 cancer cell lines, especially at higher concentrations. This broad cytotoxic response, which also extended to non-cancerous ARPE19 cells, suggests that ethanol efficiently extracts mid-to-low polarity phytochemicals (including carotenoids, essential oils, and fatty acids) capable of inducing substantial cellular stress. *C. maritimum* is well-documented to be rich in a diverse array of bioactive constituents, encompassing both hydrophilic compounds (such as polyphenols and vitamin C) and lipophilic components [41]. Such compounds are known to interact with lipid-rich cellular structures, and their ability to disrupt membrane integrity or activate apoptotic signaling pathways likely accounts for the marked reductions in viability observed in all cell types. The ethyl acetate extract exhibited selective cytotoxicity against cancer cells (HT-29 and AR42J) relative to ARPE19 normal cells, whereas the ethanol and water extracts did not show such selectivity (Table 3). This pattern suggests that, while ethyl acetate may also extract lipophilic constituents, their concentration or bioactivity may be lower than in the ethanol extract, resulting in the enhanced survival of normal cells. The ethyl acetate extract of *C. maritimum* was previously shown to induce cell death of hepatocellular carcinoma cells (HCC and Huh cells); however, those studies did not evaluate its selectivity for hepatocellular cancer cells compared to normal hepatic cells [16,42]. In contrast, the aqueous extract demonstrated minimal cytotoxic effects across all tested concentrations, with cell viability consistently exceeding 80%. This suggests that hydrophilic phytochemicals extracted with water exert limited influence on cell survival, at least in the cell lines tested. Although aqueous extracts of halophytes often contain bioactive polysaccharides, phenolic acids, or mineral constituents, their cytotoxic potential is generally lower than that of medium-to-low-polarity fractions [43,44,45], which aligns with the results obtained in the present study. Although the aqueous extract contained low levels of target phenolic acids via HPLC, its selective cytotoxicity toward AR42J cells after 48 h likely stems from non-phenolic polar constituents (e.g., polysaccharides or polar glycosides) that act cumulatively over prolonged incubation times rather than inducing acute membranous damage. Collectively, the data indicate that the anticancer potential of *C. maritimum* is strongly dependent on its medium-to-low-polarity constituents, which warrants further chemical characterization to identify the specific compounds responsible for the observed effects. However, the lack of selectivity observed in the ethanol extract toward cancer cells underscores the need for targeted fractionation or isolation strategies before considering therapeutic applications. Future studies integrating mechanistic assays—including the evaluation of apoptosis markers, oxidative stress parameters, and cell cycle profiling—will be essential to elucidate the molecular pathways underlying the cytotoxic activity of these extracts. A limitation of this study is that the non-cancerous reference cell line (ARPE19) is not tissue-matched to the cancer models, and AR42J is a rat-derived cell line; therefore, selectivity should be confirmed in tissue-matched normal human cells.

The dual anticancer and antibacterial activities exerted by the extracts suggest that natural products are promising candidates for novel therapeutic research. This study evaluated the antimicrobial activities of *C. maritimum* extracts prepared in different solvents. The results revealed that solvent polarity is a critical determinant of biological activity [46]. The aqueous extract did not affect any microorganisms, indicating that the biologically active constituents are likely phenolic or semi-polar metabolites with limited water solubility. On the other hand, ethanol and ethyl acetate extracts exerted variable levels of inhibition against several Gram-positive and Gram-negative bacteria. The remarkably low MIC values observed against *Yersinia pseudotuberculosis* and *Candida albicans* may be associated with the rich phytochemical composition of *C. maritimum*, particularly its phenolic acids, flavonoids, terpenoids, and other bioactive secondary metabolites that have previously been reported to possess significant antimicrobial and antifungal properties [6,7]. Ethanol and ethyl acetate are known to efficiently extract semi-polar and moderately lipophilic compounds, which may explain the enhanced activity observed in these extracts compared to the aqueous extract. Previous studies have demonstrated that *C. maritimum* contains diverse antimicrobial phytoconstituents and exhibits broad-spectrum biological activity [41]. In addition, flavonoids are well-recognized for their ability to disrupt microbial membranes, interfere with energy metabolism, and inhibit essential cellular processes, which may contribute to the potent activity observed in the present study [47]. Therefore, the strong antimicrobial effect may result not only from standing-alone active compounds but probably through synergistic interactions among multiple phytochemicals present in the extracts. Nevertheless, further phytochemical characterization and bioactivity-guided fractionation studies are required to identify the bioactive compounds. Consequently, although the extracts demonstrated strong growth-inhibitory effects, it remains to be determined whether their activity is bactericidal/fungicidal or bacteriostatic/fungistatic. This limits the interpretation of the potential clinical relevance of the findings. Future studies should therefore incorporate MBC/MFC analyses, time-kill kinetics, and mechanistic investigations to better evaluate the antimicrobial potential of *C. maritimum* extracts.

## 4. Materials and Methods

### 4.1. Collection, Identification, and Drying of the C. maritimum Plant

**Figure 7:**
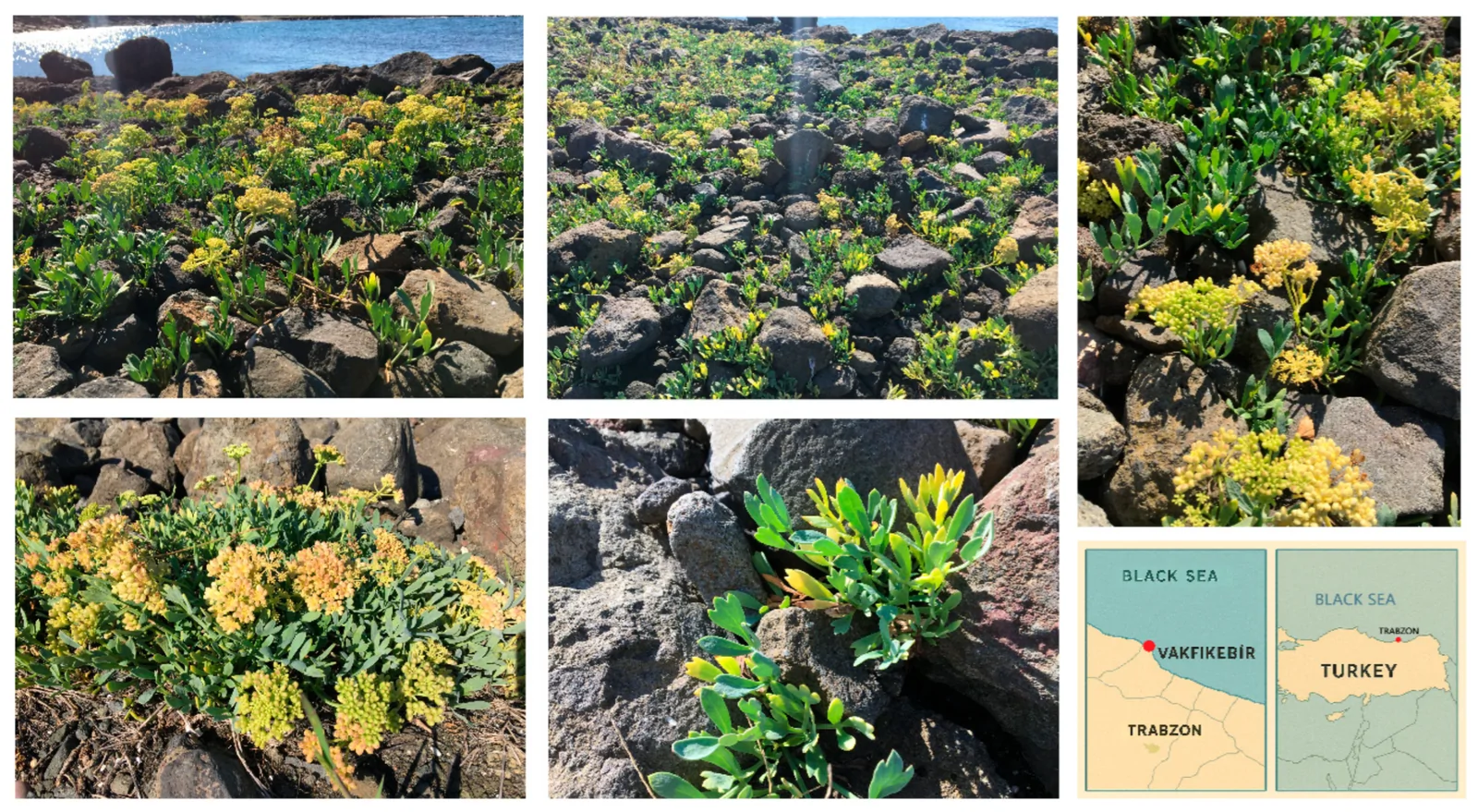
Plant sample, *C. maritimum* L., collected from coastal habitats in Yalıköy, Vakfıkebir (Trabzon Province, Türkiye).

The plant specimen belonging to the species *C. maritimum* was collected in August 2024 from Yalıköy, Vakfıkebir district, Trabzon province, with the assistance of Prof. Dr. Nevzat Batan, a faculty member of the Department of Molecular Biology and Genetics at Karadeniz Technical University (Figure 7). The plant material was carefully gathered during the flowering stage to ensure accurate identification. During the collection of plant specimens for the study, special care was taken to prevent any damage to the samples. The plants were removed from their rock substrate using appropriate tools and placed into polyethylene bags. To ensure accurate identification, specimens containing all morphological parts of the plant were collected, and the sampling location was determined and recorded using a GPS device. Collected specimens were pressed in the field using standard herbarium techniques, subsequently air-dried, and mounted on herbarium sheets. Taxonomic identification of the plant samples was performed according to “Flora of Turkey and the East Aegean Islands” [48], and voucher specimens were deposited in the Herbarium of Karadeniz Technical University (voucher number: KTUB 1238) after identification. The dried samples were then stored in cardboard boxes in a dry environment.

### 4.2. Extract Preparation

Approximately 1 kg of fresh weight of *C. maritimum* plants was collected. All plant material was roughly cleaned of dust, mud, and other plants, and then dried in the shade on paper. The dried samples were ground into a fine powder using a mechanical grinder, placed in a glass jar, and stored in a cool, dark place until use. The ethanol extract and ethyl acetate were prepared using a modified maceration method. For the ethanol extract, 10 g of powdered plant sample was placed in a 200 mL glass jar, and 100 mL (1:10, *w*/*v*) of ethanol (99.5%) was added. For the ethyl acetate extract, 100 g of powdered plant sample was placed in a 2 L glass jar, and 1 L (1:10, *w*/*v*) of ethyl acetate (99.5%) was added. Maceration was carried out for 72 h at room temperature with periodic agitation. At the end of this period, the mixtures were filtered through Whatman filter paper to separate the solvent. The resulting filtrates were pooled and concentrated under reduced pressure using a rotary evaporator to obtain the crude dry extracts. The crude ethanol and ethyl acetate extracts were dissolved in 6 mL of ethanol and 20 mL of ethyl acetate, respectively, and stored at +4 °C until further analysis [49,50,51,52]. The water extract was prepared using a modified method. 10 g of powdered plant sample was placed in a 200 mL glass jar, and 150 mL of distilled water was added (1:15, *w*/*v*). Maceration was carried out for 24 h at room temperature with periodic agitation. At the end of this period, the mixture was filtered through filter paper, and the filtrate was evaporated to dryness using a rotary evaporator, yielding a crude, dry extract. The crude water extract was dissolved in 20 mL of distilled water and stored at +4 °C until use [49,50,51,52] Table 5 shows the yield (%) of each extract. The marked variation in extraction yield between water (19.82%) and ethanol (6.5%) can be explained by the abundant presence of highly polar, non-phenolic plant components (e.g., water-soluble carbohydrates and mucilage) extracted by water. Ethanol, being a more selective solvent for moderately polar secondary metabolites, yields a lower total dry mass while potentially providing an extract enriched in specific phenolic compounds.

### 4.3. Phenolic Content of Extracts by HPLC Analysis

The analysis of phenolic content in the samples was carried out using a Shimadzu LC-20AT high-performance liquid chromatography (HPLC) system (Shimadzu Corporation, Kyoto, Japan) equipped with a photodiode array (PDA) detector and an Inertsil C18 column (250 mm × 4.6 mm, 5 μm; GL Sciences, Tokyo, Japan), following a method developed by Kara and Birinci [17]. The mobile phase consisted of eluent A (70% acetonitrile in ultrapure water, *v*/*v*) and eluent B (2% acetic acid in ultrapure water, *v*/*v*). Chromatographic separation was performed at a flow rate of 1 mL/min, an injection volume of 20 μL, and a column temperature of 30 °C.

For sample preparation, the aqueous plant extract was first adjusted to pH 2.0 using dilute hydrochloric acid (HCl), followed by liquid–liquid extraction with ethyl acetate and diethyl ether. The collected organic phases were pooled and evaporated to dryness, and the resulting residue was dissolved in absolute methanol, filtered through a 0.45 μm PTFE membrane filter, and injected into the HPLC system for analysis. Regarding the ethanolic extract, after evaporation, the remaining residue was dissolved in 10 mL of distilled water adjusted to pH 2.0 and subsequently subjected to liquid–liquid extraction with ethyl acetate and diethyl ether. The combined organic phases were evaporated to remove solvent, and the resulting residue was dissolved in 2 mL of absolute methanol, filtered through a 0.45 μm membrane filter, and analyzed. Finally, the ethyl acetate extract was also evaporated to remove the solvent; the residue was dissolved in absolute methanol, filtered through a 0.45 μm membrane filter, and injected into the HPLC system for analysis [53].

### 4.4. Cell Culture and MTT Assay for the Anticancer Activity of C. maritimum Extracts

The anticancer potential of the extracts was evaluated in two cancer cell lines (HT-29 human colon cancer cell line and AR42J rat pancreatic tumor cell line, catalog numbers HTB-38 and CRL-1492 from American Type Culture Collection (ATCC), Manassas, USA, respectively) and a healthy cell line (ARPE19 human normal retinal epithelial cells, catalog number CRL-2302 from ATCC). HT-29 and AR42J cells were selected as models of colorectal and pancreatic cancer, respectively, which represent gastrointestinal tissue samples for edible *C. maritimum*, and ARPE19 cells were used as a non-cancerous reference cell line to assess selectivity. Cells were cultured in RPMI (Capricorn Scientific GmbH, Ebsdorfergrund, Germany, Cat. No. RPMI-A) basal medium, supplemented with 10% fetal bovine serum (Biological Industries Israel Beit Haemek Ltd., Kibbutz Beit Haemek, Israel, Cat. No. 04-001-1A) and 1% penicillin-streptomycin (VivaCell Biotechnology GmbH, Denzlingen, Germany, Cat. No. C3420-0100), and incubated at 37 °C in a humidified incubator with 5% CO_2_. Before the analysis of extracts by MTT, the cytotoxicity of the solvents was evaluated (Supplementary Figure S1). Cells were seeded into 96-well plates at a density of 1 × 10^4^ viable cells per well and incubated for 24 h at 37 °C with 5% CO_2_. Afterward, the medium was replaced with fresh medium containing *C. maritimum* extracts at the desired test concentrations (2 μg/mL, 10 μg/mL, 50 μg/mL, and 250 μg/mL). Untreated cells were cultured in complete medium only. Following 24 h or 48 h incubation, the extract-containing medium was removed, and 190 μL of fresh medium along with 10 μL of MTT solution (3-(4,5-dimethylthiazol-2-yl)-2,5-diphenyl tetrazolium bromide) (Bio Basic Inc., Markham, Canada, Cat No. T0793) were added to each well and incubated for 2 h. Intracellular formazan crystal formation by metabolically active cells was confirmed microscopically, after which the MTT-containing medium was aspirated. 150 μL of dimethyl sulfoxide (DMSO) (Merck KGaA, Darmstadt, Germany, Cat No. 116743.1000) was added to each well to dissolve the formazan crystals, and the plates were incubated for 1 h at 37 °C. Absorbance was measured at 570 nm using a microplate spectrophotometer (Thermo Fisher Scientific Inc., Waltham, MA, USA, Multiskan). Cell viability in the control group (untreated cells) was set to 100%, and relative viability percentages for each treatment group were calculated. Concentration-response curves were plotted, and the half-maximal inhibitory concentration (IC50) values were calculated for cell lines exhibiting cytotoxic responses [54]. The selectivity index (SI), also known as the selectivity ratio, values were also calculated to evaluate the selective cytotoxic effect of extracts on cancer cells. It is calculated by dividing the cytotoxic concentration (IC50-normal cells) on normal cells by the effective concentration on cancer cells (IC50-cancer cells). A higher SI value indicates that an extract possesses preferential cytotoxicity toward cancer cells while remaining relatively non-toxic to normal cells. Generally, an SI value ≥ 2 is considered the benchmark threshold for a promising and selectively toxic candidate in anticancer drug discovery [55].

### 4.5. Antimicrobial Activity of C. maritimum Extracts

The antimicrobial activity of the prepared extracts was evaluated against seven bacterial species and one fungal species. The selected bacterial strains were *Bacillus subtilis* ATCC 10876, *Staphylococcus epidermidis* ATCC 12228, *Staphylococcus aureus* ATCC 25923, *Yersinia pseudotuberculosis* ATCC 911, *Escherichia coli* ATCC 25922, and *Pseudomonas aeruginosa* ATCC 27853; the fungal strain was *Candida albicans* ATCC 10231. All microbial strains were kindly provided by Prof. Dr. Hacer Muratoğlu from the Department of Molecular Biology and Genetics. To enhance the visualization of microbial growth, p-iodonitrotetrazolium violet (INT) dye was utilized as an indicator. The preparation of serial dilutions and the determination of minimum inhibitory concentration (MIC) values for each strain were performed according to established standard protocols. For the antimicrobial test, microorganisms retrieved from the −80 °C stock were subcultured on Luria Broth (LB) agar (Laboratorios Conda S.A., Torrejón de Ardoz, Spain, Cat. No. 1552). Bacteria were incubated for 16 h, and fungi for 48 h at 37 °C. Single colonies were selected and subcultured 2–3 times on fresh agar plate to obtain pure, active cultures ready prior to testing. For the experimental assays, overnight cultures of microorganisms were prepared in glass tubes in Mueller–Hinton (MHB) broth (HiMedia Laboratories Pvt. Ltd., Mumbai, India, Cat. No. M1657) and incubated at 37 °C with shaking 16 h at 220 rpm. The optical density of the growing cultures was measured at 600 nm (OD_600_). Subsequently, bacterial and fungal suspensions were adjusted to the 0.5 McFarland standard (corresponding to approximately 1.5 × 10^8^ CFU/mL) and further diluted 1:10 in sterile physiological saline (Isotonic 0.9% NaCl, Polifarma İlâç San. ve Tic. A.Ş., Tekirdağ, Türkiye). The concentrations used for all plant extracts were 0, 0.01, 0.05, 0.25, 1.25, 6.25, and 31.25 mg/mL. For each microorganism and extract type (ethanol, ethyl acetate, or distilled water), a total of 20 96-well plates (7 bacteria and 1 fungus for each plant extract) were prepared. Each well received 100 μL of MHB (Mueller Hinton Broth No. 2 Control Cations, HiMedia Laboratories Pvt. Ltd., Mumbai, India, Cat No. M1657). In the first column wells, 100 μL of the extract was added, and two-fold serial dilutions were performed across the plates. Then, 5 μL of bacterial or fungal suspension was added to each well. Positive control wells contained only the microbial inoculum and MHB, while negative control wells contained only MHB. The 96-well plates were subsequently incubated at 37 °C for 24 h for the bacterial strains and 48 h for *Candida albicans*. The same procedure was applied to each extract. Following incubation, microbial growth was visually assessed by checking for turbidity and color change upon dye addition. The minimum inhibitory concentration (MIC) was defined as the lowest concentration of the extract that completely prevented visible microbial growth [56,57,58].

### 4.6. Statistical Analyses

Cell viability percentages (%) were subjected to an angular arcsine-square root transformation to stabilize variances and satisfy the normality assumptions required for univariate analysis of variance (UNIANOVA) test using SPSS software (Version 13.0, SPSS Inc., Chicago, IL, USA) [59]. Comparisons among different concentrations within each incubation time point were performed using post-hoc analyses. Although the viability graphs display the transformed values used for UNIANOVA comparisons, the IC50 values were calculated using the original, non-transformed viability percentages. Each cell culture experiment was conducted in three independent biological replicates (using separate extract batches), with at least three technical replicates per assay run, ensuring reproducibility across extractions. Significance was defined as *p* ≤ 0.05, with significance levels indicated as follows: *p* ≤ 0.05 (*), *p* ≤ 0.01 (**), *p* ≤ 0.001 (***), and *p* ≤ 0.0001 (****). Each experiment was conducted in three independent biological replicates, with at least three technical replicates per run. Error bars represent the standard error of the mean (+/− s.e.m). Clustered heatmap and correlation matrices were generated using OriginPro software (Version 2017, OriginLab Corporation, Northampton, MA, USA).

## 5. Conclusions

Overall, the findings indicate that *C. maritimum* contains bioactive constituents with selective cytotoxicity against cancer cells, supporting its potential as a source of natural anticancer agents. However, the moderate sensitivity of non-cancerous ARPE19 cells also underscores the need for further investigation into selectivity and safety. Furthermore, several limitations of this study should be noted. First, a wider variety of non-cancerous and malignant cell lines, as well as additional microbial strains, must be tested to confirm the broader efficacy of *C. maritimum* extracts. Second, the specific bioactivities (e.g., cytotoxicity and antimicrobial effects) of each isolated compound remain to be explored. The current study focused primarily on providing a foundational baseline comparing solvent-dependent bioactivities and phenolic profiles, rather than isolating individual active molecules or dissecting specific intracellular signaling pathways. Future bioactivity-guided fractionation and detailed molecular mechanism studies (e.g., apoptosis pathways and cell cycle regulation) will be crucial to clarify the structure-dependent modes of action of *C. maritimum* active constituents. Third, although the MTT assay and morphological observations confirm concentration-dependent loss of viability and apoptotic features (e.g., cell shrinkage and detachment), the exact mode of cell death (such as caspase-dependent apoptosis, ROS-mediated cell death, or autophagy) remains to be formally characterized. Based on current literature on the identified bioactive phenolics (e.g., apigenin, gallic acid, and ferulic acid), the cytotoxicity is likely mediated via apoptotic cascades, which will be confirmed in detailed follow-up mechanistic assays. Fourth, variations in plant composition based on harvest time and geographic location should be analyzed, alongside a separate evaluation of their biological effects. Fourth, the cytotoxic effect of ethyl acetate on HT-29 cells represented another limitation. Future studies should evaluate the extracts using alternative solubilization strategies, such as concentrating the extracts and testing them at lower final solvent concentrations, or normalizing extract effects relative to their corresponding solvent controls. Finally, the molecular and intracellular mechanisms of the extracts have yet to be fully elucidated. Consequently, these limitations mean our current findings should be considered preliminary, as the total profile and exact mechanisms of the extracts cannot yet be generalized across all conditions. In this context, however, our study successfully establishes a foundational baseline, concluding that *C. maritimum* possesses significant potential against pancreatic and colon cancers. Thus, despite these boundaries, the data firmly position *C. maritimum* as a prominent candidate for detailed, downstream biological and pharmacological studies. Future studies should therefore focus on identifying the specific compounds responsible for these effects and on elucidating their mechanisms of action through flow cytometry, ROS quantification, and apoptosis-related molecular assays.

## Figures and Tables

**Table 1:** Biochemical Profiles of *C. maritimum* Extracts.

Phenolic Content (µg Phenolic/g)		Extracts		
		dH_2_O	EtOH	EtAc
**Phenolic Acids**	**Hydroxybenzoic acids**			
	p-OH Benzoic acid	N.D	56.40	16.72
	Protocatechuic acid	175.64	267.30	57.73
	Gallic acid	33.35	44.67	1.79
	**Hydroxycinnamic acids**			
	Ferulic acid	N.D	196.26	36.45
	p-Coumaric acid	N.D	91.48	30.21
**Flavonoids**	**Flavan-3-ols**			
	Apigenin	N.D	14.53	3.66
	**Flavanones**			
	Pinocembrin	N.D	18.93	11.85

N.D., not detected, Note: A total of 26 phenolic standards were screened via HPLC. Only compounds detected in at least one extract fraction are presented in Table 1. The full analytical panel of all 26 standards, including compounds below the limit of detection across all extracts, is provided in Supplementary Table S1.

**Table 2:** Average IC50 values (µg/mL) +/− s.e.m. in ARPE19, HT-29, and AR42J cell lines after treatments with ethanol, distilled water, and ethyl acetate extracts of *C. maritimum.* Note: Vehicle controls corresponding to the solvent proportions present in the extract dilutions (up to 0.03% *v*/*v* for ethyl acetate at 250 µg/mL) were tested concurrently and showed no significant cytotoxicity (Figure S1). Raw IC50 and SI values were calculated relative to untreated control cells.

**Extracts**	**Incubation (h)**	**Cells**					
		**ARPE19**		**HT-29**		**AR42J**	
		**24 h**	**48 h**	**24 h**	**48 h**	**24 h**	**48 h**
	Ethanol	65.5 ± 3.1	55.1 ± 4.3	88 ± 5.3	72.1 ± 3.2	54.8 ± 3	65.4 ± 3.4
	Distilled Water	>250	>250	>250	>250	>250	95 ± 12.4
	Ethyl Acetate	>250	>250	>250	>250	82.6 ± 2.1	78.7 ± 2.24

**Table 3:** Average selective indices (SI) of extracts for each cancer cell.

Extract Using	HT-29 Colon Cancer		AR42J Pancreas Cancer	
	24 h	48 h	24 h	48 h
Ethanol	0.744	0.764	1.195	0.843
Distilled Water	≈1	≈1	≈1	2.632
Ethyl Acetate	≈1	≈1	3.027	3.177

Note: Vehicle controls corresponding to the solvent proportions present in the extract dilutions (up to 0.03% *v*/*v* for ethyl acetate at 250 µg/mL) were tested concurrently and showed no significant cytotoxicity (Figure S1). Raw IC50 and SI values were calculated relative to untreated control cells.

**Table 4:** Antimicrobial results of *C. maritimum* extracts.

Microorganism	MIC Values of Sea Fennel Extracts (mg/mL)			Positive Control
	Ethanol	Ethyl Acetate	Water	Ampicillin (100 µg/mL)
*B. subtilis*	12.50	>31.25	N.E	+
*S. epidermidis*	>31.25	>31.25	N.E	+
*S. aureus*	6.125	6.125	N.E	+
*K. pneumonia*	>31.25	>31.25	N.E	+
*Y. pseudotuberculosis*	>31.25	0.01	N.E	+
*E. coli*	>31.25	>31.25	N.E	+
*P. aeruginosa*	N.E.	>31.25	N.E	+
*C. albicans*	0.01	0.01	N.E	+

MIC values were shown as mg/mL. MIC values of some extracts could not be determined and were expressed as ‘No Effect’ (N.E) in the Table. **+**: Complete inhibition was observed at the tested concentration.

**Table 5:** Percent (%) yield calculation of *C. maritimum* extracts.

Solvent	Amount of Ground Sample	Yield	Yield Percentage
Ethanol (CH_3_CH_2_OH)	10 g	0.605 g	6.5%
Water (H_2_O)	10 g	1.982 g	19.82%
Ethyl acetate (CH_3_COOC_2_H_5_)	100 g	10 g	10%

## Data Availability

The data that support the findings of this study are available in the Supplementary Materials of this article.

## Supplementary Materials

The following supporting information can be downloaded at https://www.mdpi.com/article/10.3390/molecules31213877/s1, Figure S1: Cell Viability (%) after solvent exposure for 24 h and 48 h in AR42J, HT-29 and ARPE19 cells; Figure S2: Post-Hoc analyses of cell viability for dose comparisons after different extracts in three cells, Figure S3: INT colorimetric assay plates, Figure S4: Antimicrobial activity of solvents, Table S1: HPLC analysis of *Crithmum maritimum* extracts by distilled water, ethanol (EtOH) and ethyl acetate (EtAc), Table S2: Antimicrobial results of ethyl acetate, water and ethanol solvent.

## References

[1] Sawicki T., Ruszkowska M., Danielewicz A., Niedźwiedzka E., Arłukowicz T., Przybyłowicz K.E. (2021). A Review of Colorectal Cancer in Terms of Epidemiology, Risk Factors, Development, Symptoms and Diagnosis. Cancers.

[2] Bray F., Laversanne M., Sung H., Ferlay J., Siegel R.L., Soerjomataram I., Jemal A. (2024). Global Cancer Statistics 2022: GLOBOCAN Estimates of Incidence and Mortality Worldwide for 36 Cancers in 185 Countries. CA Cancer J. Clin..

[3] Souid A., Della Croce C.M., Frassinetti S., Gabriele M., Pozzo L., Ciardi M., Abdelly C., Ben Hamed K., Magné C., Longo V. (2021). Nutraceutical Potential of Leaf Hydro-Ethanolic Extract of the Edible Halophyte *Crithmum maritimum* L. Molecules.

[4] Kraouia M., Nartea A., Maoloni A., Osimani A., Garofalo C., Fanesi B., Ismaiel L., Aquilanti L., Pacetti D. (2023). Sea Fennel (*Crithmum maritimum* L.) as an Emerging Crop for the Manufacturing of Innovative Foods and Nutraceuticals. Molecules.

[5] Alemán A., Marín D., Taladrid D., Montero P., Carmen Gómez-Guillén M. (2019). Encapsulation of Antioxidant Sea Fennel (*Crithmum maritimum*) Aqueous and Ethanolic Extracts in Freeze-Dried Soy Phosphatidylcholine Liposomes. Food Res. Int..

[6] Correia I., Antunes M., Tecelão C., Neves M., Pires C.L., Cruz P.F., Rodrigues M., Peralta C.C., Pereira C.D., Reboredo F. (2024). Nutritive Value and Bioactivities of a Halophyte Edible Plant: *Crithmum maritimum* L. (Sea Fennel). Plants.

[7] Nabet N., Boudries H., Chougui N., Loupassaki S., Souagui S., Burló F., Hernández F., Carbonell-Barrachina Á.A., Madani K., Larbat R. (2017). Biological Activities and Secondary Compound Composition from *Crithmum maritimum* Aerial Parts. Int. J. Food Prop..

[8] Dzhoglova V., Ivanov K., Benbassat N., Georgieva-Dimova Y., Ardasheva R., Karcheva-Bahchevanska D., Ivanova S. (2024). *Crithmum maritimum* L.—Study on the Histochemical Localization of Essential Oil. Plants.

[9] Renna M. (2018). Reviewing the Prospects of Sea Fennel (*Crithmum maritimum* L.) as Emerging Vegetable Crop. Plants.

[10] Atia A., Barhoumi Z., Mokded R., Abdelly C., Smaoui A. (2011). Environmental Eco-Physiology and Economical Potential of the Halophyte *Crithmum maritimum* L. (Apiaceae). J. Med. Plants Res..

[11] Pedreiro S., Figueirinha A., Cavaleiro C., Cardoso O., Donato M.M., Salgueiro L., Ramos F. (2023). Exploiting the *Crithmum maritimum* L. Aqueous Extracts and Essential Oil as Potential Preservatives in Food, Feed, Pharmaceutical and Cosmetic Industries. Antioxidants.

[12] Ashim A., Ismaiel L., Fanesi B., Nartea A., Maoloni A., Orhotohwo O.L., Darko H.S.O., Lucci P., Aquilanti L., Pacetti D. (2025). Food-Grade Polar Extracts from Sea Fennel (*Crithmum maritimum* L.) By-Products: Unlocking Potential for the Food Industry. Foods.

[13] Pereira C.G., Barreira L., da Rosa Neng N., Nogueira J.M.F., Marques C., Santos T.F., Varela J., Custódio L. (2017). Searching for New Sources of Innovative Products for the Food Industry within Halophyte Aromatic Plants: In Vitro Antioxidant Activity and Phenolic and Mineral Contents of Infusions and Decoctions of *Crithmum maritimum* L. Food Chem. Toxicol..

[14] Kumarasamy Y., Cox P.J., Jaspars M., Nahar L., Dey Sarker S. (2002). Screening Seeds of Scottish Plants for Antibacterial Activity. J. Ethnopharmacol..

[15] Gnocchi D., Nikolic D., Paparella R.R., Sabbà C., Mazzocca A. (2024). *Crithmum maritimum* Extract Restores Lipid Homeostasis and Metabolic Profile of Liver Cancer Cells to a Normal Phenotype. Plant Foods Hum. Nutr..

[16] Gnocchi D., Sabbà C., Mazzocca A. (2022). The Edible Plant *Crithmum maritimum* Shows Nutraceutical Properties by Targeting Energy Metabolism in Hepatic Cancer. Plant Foods Hum. Nutr..

[17] Kara Y., Birinci C. (2024). Usability of the Phenolic Profile Analysis Method Developed in RP-HPLC-PDA in Natural Products. J. Apitherapy Nat..

[18] Martins-Noguerol R., Matías L., Pérez-Ramos I.M., Moreira X., Muñoz-Vallés S., Mancilla-Leytón J.M., Francisco M., García-González A., DeAndrés-Gil C., Martínez-Force E. (2022). Differences in Nutrient Composition of Sea Fennel (*Crithmum maritimum*) Grown in Different Habitats and Optimally Controlled Growing Conditions. J. Food Compos. Anal..

[19] Jang J.Y., Sung B., Kim N.D. (2022). Role of Induced Programmed Cell Death in the Chemopreventive Potential of Apigenin. Int. J. Mol. Sci..

[20] Ahmadzadeh K., Roshdi Dizaji S., Ramezani F., Imani F., Shamseddin J., Sarveazad A., Yousefifard M. (2024). Potential Therapeutic Effects of Apigenin for Colorectal Adenocarcinoma: A Systematic Review and Meta-Analysis. Cancer Med..

[21] Amini P., Moazamiyanfar R., Dakkali M.S., Jafarzadeh E., Ganjizadeh M., Rastegar-Pouyani N., Moloudi K., Khodamoradi E., Taeb S., Najafi M. (2023). Induction of Cancer Cell Death by Apigenin: A Review on Different Cell Death Pathways. Mini Rev. Med. Chem..

[22] Ghanbari-Movahed M., Shafiee S., Burcher J.T., Lagoa R., Farzaei M.H., Bishayee A. (2023). Anticancer Potential of Apigenin and Isovitexin with Focus on Oncogenic Metabolism in Cancer Stem Cells. Metabolites.

[23] Elbatreek M.H., Mahdi I., Ouchari W., Mahmoud M.F., Sobeh M. (2023). Current Advances on the Therapeutic Potential of Pinocembrin: An Updated Review. Biomed. Pharmacother..

[24] Singla N., Mittal P., Babu M.A., Menon S.V., Ray S., Ali H., Purohit M., Goyal K., Mishra R., Hussain M.S. (2025). Pinocembrin as a Novel Anti-Cancer Agent: Exploring Preclinical Evidence along with Therapeutic Potential. World J. Exp. Med..

[25] Abdullah H., Ismail I., Suppian R., Zakaria N.M. (2023). Natural Gallic Acid and Methyl Gallate Induces Apoptosis in Hela Cells through Regulation of Intrinsic and Extrinsic Protein Expression. Int. J. Mol. Sci..

[26] Hassani S., Ghanbari F., Lotfi M., Alam W., Aschner M., Popović-Djordjević J., Shahcheraghi S.H., Khan H. (2023). How Gallic Acid Regulates Molecular Signaling: Role in Cancer Drug Resistance. Med. Oncol..

[27] Keyvani-Ghamsari S., Rahimi M., Khorsandi K. (2023). An Update on the Potential Mechanism of Gallic Acid as an Antibacterial and Anticancer Agent. Food Sci. Nutr..

[28] Cadena-Iñiguez J., Santiago-Osorio E., Sánchez-Flores N., Salazar-Aguilar S., Soto-Hernández R.M., Riviello-Flores M.d.l.L., Macías-Zaragoza V.M., Aguiñiga-Sánchez I. (2024). The Cancer-Protective Potential of Protocatechuic Acid: A Narrative Review. Molecules.

[29] Kalinowska M., Gołębiewska E., Świderski G., Męczyńska-Wielgosz S., Lewandowska H., Pietryczuk A., Cudowski A., Astel A., Świsłocka R., Samsonowicz M. (2021). Plant-Derived and Dietary Hydroxybenzoic Acids—A Comprehensive Study of Structural, Anti-/pro-Oxidant, Lipophilic, Antimicrobial, and Cytotoxic Activity in Mda-Mb-231 and Mcf-7 Cell Lines. Nutrients.

[30] Myint O., Wattanapongpitak S., Kothan S., Udomtanakunchai C., Tima S., Tungjai M. (2022). Modulation of P-Glycoprotein-Mediated Efflux Pirarubicin in Living Multidrug-Resistant K562/Dox Cell Lines by 4-Hydroxybenzoic Acid and 4-Hydroxy-3-Methoxybenzoic Acid via Impairment of the Cellular Energetic State. Toxicol. Rep..

[31] Bao X., Li W., Jia R., Meng D., Zhang H., Xia L. (2023). Molecular Mechanism of Ferulic Acid and Its Derivatives in Tumor Progression. Pharmacol. Rep..

[32] Purushothaman J.R., Rizwanullah M. (2024). Ferulic Acid: A Comprehensive Review. Cureus.

[33] Singh Tuli H., Kumar A., Ramniwas S., Coudhary R., Aggarwal D., Kumar M., Sharma U., Chaturvedi Parashar N., Haque S., Sak K. (2022). Ferulic Acid: A Natural Phenol That Inhibits Neoplastic Events through Modulation of Oncogenic Signaling. Molecules.

[34] Tehami W., Nani A., Khan N.A., Hichami A. (2023). New Insights Into the Anticancer Effects of P-Coumaric Acid: Focus on Colorectal Cancer. Dose Response.

[35] Hoveizi E., Hushmandi K. (2024). Comparison of Effects of P-Coumaric Acid and Coumarin on Colorectal Cancer Cell Line by Inducing Apoptosis and Autophagy. Avicenna J. Phytomed..

[36] Cui K., Wu H., Fan J., Zhang L., Li H., Guo H., Yang R., Li Z. (2022). The Mixture of Ferulic Acid and P-Coumaric Acid Suppresses Colorectal Cancer through LncRNA 495810/PKM2 Mediated Aerobic Glycolysis. Int. J. Mol. Sci..

[37] Xiao K., Zhou M., Lv Q., He P., Qin X., Wang D., Zhao J., Liu Y. (2023). Protocatechuic Acid and Quercetin Attenuate ETEC-Caused IPEC-1 Cell Inflammation and Injury Associated with Inhibition of Necroptosis and Pyroptosis Signaling Pathways. J. Anim. Sci. Biotechnol..

[38] Crowe-White K.M., Evans L.W., Kuhnle G.G.C., Milenkovic D., Stote K., Wallace T., Handu D., Senkus K.E. (2022). Flavan-3-Ols and Cardiometabolic Health: First Ever Dietary Bioactive Guideline. Adv. Nutr..

[39] Luo Y., Jian Y., Liu Y., Jiang S., Muhammad D., Wang W. (2022). Flavanols from Nature: A Phytochemistry and Biological Activity Review. Molecules.

[40] Baschiera E., Sorrentino U., Calderan C., Desbats M.A., Salviati L. (2021). The Multiple Roles of Coenzyme Q in Cellular Homeostasis and Their Relevance for the Pathogenesis of Coenzyme Q Deficiency. Free Radic. Biol. Med..

[41] Dzhoglova V., Ivanova S., Shishmanova-Doseva M., Saracheva K. (2025). *Crithmum maritimum* L.: Phytochemical Profile, Biological Activities, and Therapeutic Potential. Molecules.

[42] Gnocchi D., Cesari G., Calabrese G.J., Capone R., Sabbà C., Mazzocca A. (2020). Inhibition of Hepatocellular Carcinoma Growth by Ethyl Acetate Extracts of Apulian *Brassica oleracea* L. and *Crithmum maritimum* L. Plant Foods Hum. Nutr..

[43] Hawas U.W., El-Kassem L.T.A., Shaher F.M., Al-Farawati R., Ghandourah M. (2022). Phytochemical Compositions of Some Red Sea Halophyte Plants with Antioxidant and Anticancer Potentials. Molecules.

[44] Rodrigues M.J., Jekő J., Cziáky Z., Pereira C.G., Custódio L. (2022). The Medicinal Halophyte *Frankenia laevis* L. (Sea Heath) Has In Vitro Antioxidant Activity, α-Glucosidase Inhibition, and Cytotoxicity towards Hepatocarcinoma Cells. Plants.

[45] Rodrigues M.J., Gangadhar K.N., Vizetto-Duarte C., Wubshet S.G., Nyberg N.T., Barreira L., Varela J., Custódio L. (2014). Maritime Halophyte Species from Southern Portugal as Sources of Bioactive Molecules. Mar. Drugs.

[46] Tian F., Li B., Ji B., Yang J., Zhang G., Chen Y., Luo Y. (2009). Antioxidant and Antimicrobial Activities of Consecutive Extracts from Galla Chinensis: The Polarity Affects the Bioactivities. Food Chem..

[47] Górniak I., Bartoszewski R., Króliczewski J. (2019). Comprehensive Review of Antimicrobial Activities of Plant Flavonoids. Phytochem. Rev..

[48] Davis P.H. (1972). Flora of Turkey and the East Aegean Islands.

[49] Yildirim Akatin M., Er Kemal M., Batan N. (2022). Antimicrobial Activities of Some Bryophytes Collected from Trabzon, Türkiye and Preparation of Herbal Soap and Cream Using Pellia Epiphylla Extract for the First Time. Anatol. Bryol..

[50] Korkmaz N., Dayangaç A., Sevindik M. (2021). Antioxidant, Antimicrobial and Antiproliferative Activities of Galium Aparine. Ank. Univ. Eczaci. Fak. Derg..

[51] Erik İ., Yaylı N., Coşkunçelebi K., Makbul S., Karaoğlu Ş.A. (2021). Three New Dihydroisocoumarin Glycosides with Antimicrobial Activities from Scorzonera Aucheriana. Phytochem. Lett..

[52] Erik İ., Coşkunçelebi K., Makbul S., Yayli N. (2021). New Chlorogenic Acid Derivatives and Triterpenoids from Scorzonera Aucheriana. Turk. J. Chem..

[53] Kara Y., Can Z., Kolaylı S. (2022). Applicability of Phenolic Profile Analysis Method Developed with RP-HPLC-PDA to Some Bee Product. Braz. Arch. Biol. Technol..

[54] Ayazoglu Demir E., Colak A., Celik Uzuner S., Bekircan O. (2021). Cytotoxic Effect of a 3-(4-Chlorophenyl)-5-(4-Methoxybenzyl)-4H-1,2,4-Triazole Derivative Compound in Human Melanoma Cells. Int. J. Biol. Chem..

[55] de Oliveira P.F., Alves J.M., Damasceno J.L., Oliveira R.A.M., Júnior Dias H., Crotti A.E.M., Tavares D.C. (2015). Cytotoxicity Screening of Essential Oils in Cancer Cell Lines. Rev. Bras. Farmacogn..

[56] Weseler A., Geiss H.K., Saller R., Reichling J. (2005). A Novel Colorimetric Broth Microdilution Method to Determine the Minimum Inhibitory Concentration (MIC) of Antibiotics and Essential Oils against *Helicobacter pylori*. Pharmazie.

[57] Süleymanoğlu N., Ünver Y., Ustabaş R., Çelik F., Güler H.İ., Direkel Ş., Bektaş K.İ. (2024). Synthesis, Characterization, and Biological Activity of Novel 1,2,4-Triazole Moieties: Antileishmania, Antimicrobial Effects, and Molecular Docking Studies. J. Mol. Struct..

[58] Nouayti F.Z., Oufkir N., Lahmidi S., Rehioui M., Anouar E.H., Filali I., Hajji H., El Yazidi M., Mague J.T., Essassi E.M. (2025). Synthesis, X-Ray, Spectroscopic Characterization (NMR, FT-IR, Raman, and UV–VIS), Hirshfeld Surface Analysis, DFT, Molecular Docking, and Estimating Anticancer of 5,6-Diphenyl-3-(Prop-2-Yn-1-Ylthio)-1,2,4-Triazine. J. Mol. Struct..

[59] Campbell R.C., Sokal R.R., Rohlf F.J. (1970). Biometry: The Principles and Practice of Statistics in Biological Research. J. R. Stat. Soc. Ser. A.

