# Supplementary material for: Polarity-Guided Extraction of Crithmum maritimum L.: Phenolic Profiling, Selective Anticancer Activity in AR42J and HT-29 Cells, and Antimicrobial Effects
Source: Molecules. 2026 Oct 27;31(21):3877. doi: 10.3390/molecules31213877 (PMC13765143; doi:10.3390/molecules31213877)
Supplement: Supplementary file 1 [file molecules-31-03877-s001.zip › molecules-4621793-supplementary.pdf]

Polarity-Guided Extraction of *Crithmum maritimum L.*: Phenolic Profiling, Selective Anticancer Activity in AR42J and HT-29 Cells, and Antimicrobial Effects

SUPPLEMENTARY DATA

Table S1. HPLC analysis of *Crithmum maritimum* extracts by distilled water, ethanol (EtOH) and ethyl acetate (EtAc)

Tests		Extracts		
		Distilled Water	EtOH	EtAc
	**Phenolic Standards (µg phenolic/g)**			
Phenolic acids	***Hydroxybenzoic acids***			
	p-OH Benzoic acid	-	56,402	16,718
	Protocathehuic acid	175,643	267,301	57,734
	Gallic acid	33,348	44,669	1,792
	Chlorogenic acid	-	-	-
	Syringic acid	-	-	-
	Ellagic acid	-	-	-
	Vanillic acid	-	-	-
	Hydroxycinnamic acids			
	t-cinnamic acid	-	-	-
	Ferulic acid	-	196,261	36,454
	p-Coumaric acid	-	91,481	30,210
	Caffeic acid	-	-	-
	Caffeic acid phenethyl ester (CAPE)	-	-	-
**Flavonoids**	***Flavanol***			
	Rhamnetin	-	-	-
	Quercetin	-	-	-
	Rutin	-	-	-
	Myricetin	-	-	-
	Galangin	-	-	-
	Flavan-3-ols			
	Epicatechin	-	-	-
	Catechin hydrate	-	-	-
	Flavones			
	Chrysin	-	-	-
	Daidzein	-	-	-
	Apigenin	-	14,519	3,658
	Luteolin	-	-	-
	Flavanones			
	Pinocembrin	-	18,933	11,852
	Hesperetin	-	-	-
	Naringenin	-	-	-

-: not detected

Figure S1. Cell Viability (%) after solvent exposure for 24h and 48h in AR42J, HT-29 and ARPE19 cells

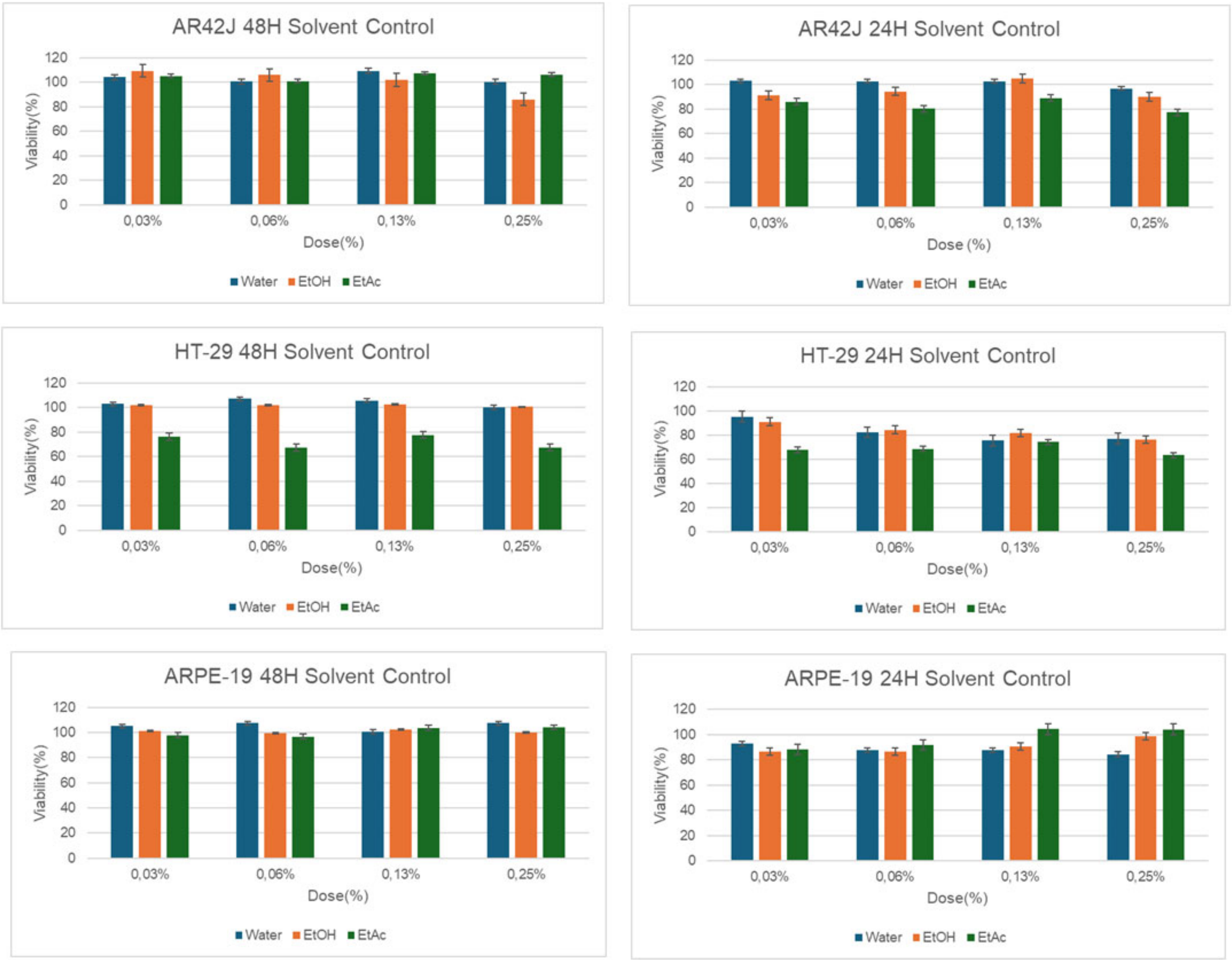

Figure S2. Post-Hoc analyses of cell viability for dose comparisons after different extracts in three cells

AR42J

Ethanol — 24

(I) Dose	(J) Dose	Sig.
,00	2,00	,006
	10,00	,002
	50,00	,000
	250,00	,000
2,00	,00	,006
	10,00	,583
	50,00	,000
	250,00	,000
10,00	,00	,002
	2,00	,583
	50,00	,000
	250,00	,000
50,00	,00	,000
	2,00	,000
	10,00	,000
	250,00	,000
250,00	,00	,000
	2,00	,000
	10,00	,000
	50,00	,000

Ethanol — 48

(I) Dose	(J) Dose	Sig.
,00	2,00	,034
	10,00	,569
	50,00	,004
	250,00	,000
2,00	,00	,034
	10,00	,109
	50,00	,349
	250,00	,000
10,00	,00	,569
	2,00	,109
	50,00	,015
	250,00	,000
50,00	,00	,004
	2,00	,349
	10,00	,015
	250,00	,000
250,00	,00	,000
	2,00	,000
	10,00	,000
	50,00	,000

Distilled Water — 24

(I) Dose	(J) Dose	Sig.
,00	2,00	,048
	10,00	,030
	50,00	,038
	250,00	,037
2,00	,00	,048
	10,00	,820
	50,00	,914
	250,00	,897
10,00	,00	,030
	2,00	,820
	50,00	,905
	250,00	,922
50,00	,00	,038
	2,00	,914
	10,00	,905
	250,00	,984
250,00	,00	,037
	2,00	,897
	10,00	,922
	50,00	,984

Distilled Water — 48

(I) Dose	(J) Dose	Sig.
,00	2,00	,748
	10,00	,423
	50,00	,017
	250,00	,018
2,00	,00	,748
	10,00	,628
	50,00	,034
	250,00	,036
10,00	,00	,423
	2,00	,628
	50,00	,092
	250,00	,096
50,00	,00	,017
	2,00	,034
	10,00	,092
	250,00	,981
250,00	,00	,018
	2,00	,036
	10,00	,096
	50,00	,981

Ethyl Acetate — 24

(I) Dose	(J) Dose	Sig.
,00	2,00	,521
	10,00	1,000
	50,00	,043
	250,00	,000
2,00	,00	,521
	10,00	,521
	50,00	,152
	250,00	,000
10,00	,00	1,000
	2,00	,521
	50,00	,043
	250,00	,000
50,00	,00	,043
	2,00	,152
	10,00	,043
	250,00	,000
250,00	,00	,000
	2,00	,000
	10,00	,000
	50,00	,000

Ethyl Acetate — 48

(I) Dose	(J) Dose	Sig.
,00	2,00	,304
	10,00	,194
	50,00	,137
	250,00	,000
2,00	,00	,304
	10,00	,778
	50,00	,630
	250,00	,000
10,00	,00	,194
	2,00	,778
	50,00	,842
	250,00	,000
50,00	,00	,137
	2,00	,630
	10,00	,842
	250,00	,000
250,00	,00	,000
	2,00	,000
	10,00	,000
	50,00	,000

ARPE-19

Ethanol — 24

(I) Dose	(J) Dose	Sig.
,00	2,00	,057
	10,00	,013
	50,00	,009
	250,00	,000
2,00	,00	,057
	10,00	,509
	50,00	,419
	250,00	,000
10,00	,00	,013
	2,00	,509
	50,00	,880
	250,00	,000
50,00	,00	,009
	2,00	,419
	10,00	,880
	250,00	,000
250,00	,00	,000
	2,00	,000
	10,00	,000
	50,00	,000

Ethanol — 48

(I) Dose	(J) Dose	Sig.
,00	2,00	,012
	10,00	,003
	50,00	,000
	250,00	,000
2,00	,00	,012
	10,00	,576
	50,00	,027
	250,00	,000
10,00	,00	,003
	2,00	,576
	50,00	,087
	250,00	,001
50,00	,00	,000
	2,00	,027
	10,00	,087
	250,00	,080
250,00	,00	,000
	2,00	,000
	10,00	,001
	50,00	,080

Distilled Water — 24

(I) Dose	(J) Dose	Sig.
,00	2,00	,037
	10,00	,003
	50,00	,004
	250,00	,015
2,00	,00	,037
	10,00	,311
	50,00	,347
	250,00	,678
10,00	,00	,003
	2,00	,311
	50,00	,941
	250,00	,545
50,00	,00	,004
	2,00	,347
	10,00	,941
	250,00	,595
250,00	,00	,015
	2,00	,678
	10,00	,545
	50,00	,595

Distilled Water — 48

(I) Dose	(J) Dose	Sig.
,00	2,00	,041
	10,00	,014
	50,00	,004
	250,00	,228
2,00	,00	,041
	10,00	,622
	50,00	,339
	250,00	,368
10,00	,00	,014
	2,00	,622
	50,00	,639
	250,00	,169
50,00	,00	,004
	2,00	,339
	10,00	,639
	250,00	,070
250,00	,00	,228
	2,00	,368
	10,00	,169
	50,00	,070

Ethyl Acetate — 24

(I) Dose	(J) Dose	Sig.
,00	2,00	,418
	10,00	,244
	50,00	,023
	250,00	,012
2,00	,00	,418
	10,00	,715
	50,00	,121
	250,00	,073
10,00	,00	,244
	2,00	,715
	50,00	,227
	250,00	,146
50,00	,00	,023
	2,00	,121
	10,00	,227
	250,00	,794
250,00	,00	,012
	2,00	,073
	10,00	,146
	50,00	,794

Ethyl Acetate — 48

(I) Dose	(J) Dose	Sig.
,00	2,00	,253
	10,00	,072
	50,00	,000
	250,00	,000
2,00	,00	,253
	10,00	,487
	50,00	,000
	250,00	,000
10,00	,00	,072
	2,00	,487
	50,00	,000
	250,00	,000
50,00	,00	,000
	2,00	,000
	10,00	,000
	250,00	,967
250,00	,00	,000
	2,00	,000
	10,00	,000
	50,00	,967

HT-29

Ethanol — 24

(I) Dose	(J) Dose	Sig.
,00	2,00	,038
	10,00	,534
	50,00	,232
	250,00	,000
2,00	,00	,038
	10,00	,130
	50,00	,341
	250,00	,000
10,00	,00	,534
	2,00	,130
	50,00	,559
	250,00	,000
50,00	,00	,232
	2,00	,341
	10,00	,559
	250,00	,000
250,00	,00	,000
	2,00	,000
	10,00	,000
	50,00	,000

Ethanol — 48

(I) Dose	(J) Dose	Sig.
,00	2,00	,128
	10,00	,156
	50,00	,005
	250,00	,000
2,00	,00	,128
	10,00	,912
	50,00	,151
	250,00	,000
10,00	,00	,156
	2,00	,912
	50,00	,124
	250,00	,000
50,00	,00	,005
	2,00	,151
	10,00	,124
	250,00	,000
250,00	,00	,000
	2,00	,000
	10,00	,000
	50,00	,000

Distilled Water — 24

(I) Dose	(J) Dose	Sig.
,00	2,00	,264
	10,00	,409
	50,00	,443
	250,00	,178
2,00	,00	,264
	10,00	,764
	50,00	,720
	250,00	,810
10,00	,00	,409
	2,00	,764
	50,00	,953
	250,00	,590
50,00	,00	,443
	2,00	,720
	10,00	,953
	250,00	,550
250,00	,00	,178
	2,00	,810
	10,00	,590
	50,00	,550

Distilled Water — 48

(I) Dose	(J) Dose	Sig.
,00	2,00	,143
	10,00	,647
	50,00	1,000
	250,00	1,000
2,00	,00	,143
	10,00	,304
	50,00	,143
	250,00	,143
10,00	,00	,647
	2,00	,304
	50,00	,647
	250,00	,647
50,00	,00	1,000
	2,00	,143
	10,00	,647
	250,00	1,000
250,00	,00	1,000
	2,00	,143
	10,00	,647
	50,00	1.000

Ethyl Acetate — 24

(I) Dose	(J) Dose	Sig.
,00	2,00	,190
	10,00	,327
	50,00	,008
	250,00	,000
2,00	,00	,190
	10,00	,732
	50,00	,137
	250,00	,008
10,00	,00	,327
	2,00	,732
	50,00	,071
	250,00	,004
50,00	,00	,008
	2,00	,137
	10,00	,071
	250,00	,196
250,00	,00	,000
	2,00	,008
	10,00	,004
	50,00	,196

Ethyl Acetate — 48

(I) Dose	(J) Dose	Sig.
,00	2,00	,150
	10,00	,022
	50,00	,025
	250,00	,000
2,00	,00	,150
	10,00	,352
	50,00	,373
	250,00	,000
10,00	,00	,022
	2,00	,352
	50,00	,967
	250,00	,004
50,00	,00	,025
	2,00	,373
	10,00	,967
	250,00	,003
250,00	,00	,000
	2,00	,000
	10,00	,004
	50,00	,003

Figure S3.INT colorimetric assay plates

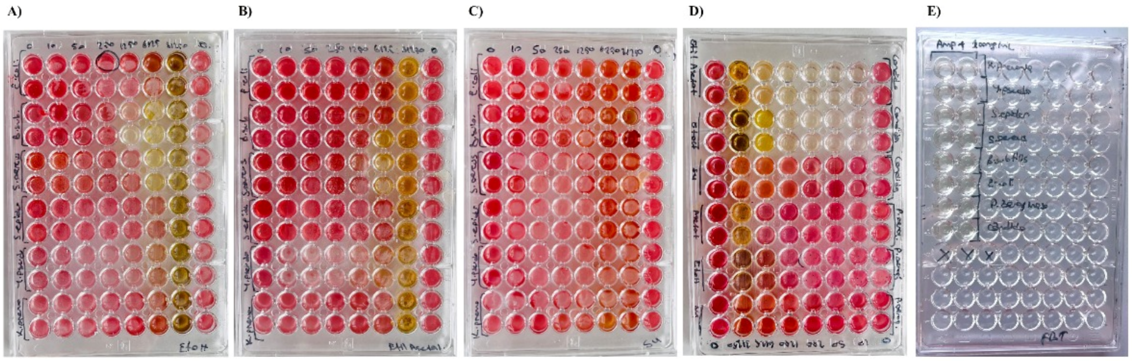

INT colorimetric assay. MIC doses of A) Ethanol solvent extract in all of bacteria except *P. aeroginosa*, B) Ethyl acetate solvent extract in all of bacteria except *P. aeroginosa*, C) Water solvent extract in all of bacteria except *P. aeroginosa*, D) Ethanol, ethyl acetate and water extract solvent extract in fungi and *P. aeruginosa,* E) Positive control ampicillin (100µg/ml) in all of bacteria and fungi.

Figure S4. Antimicrobial activity of solvents

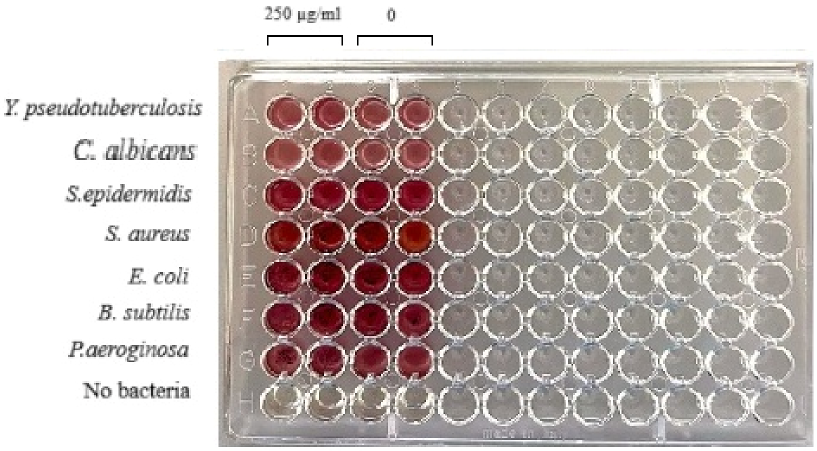

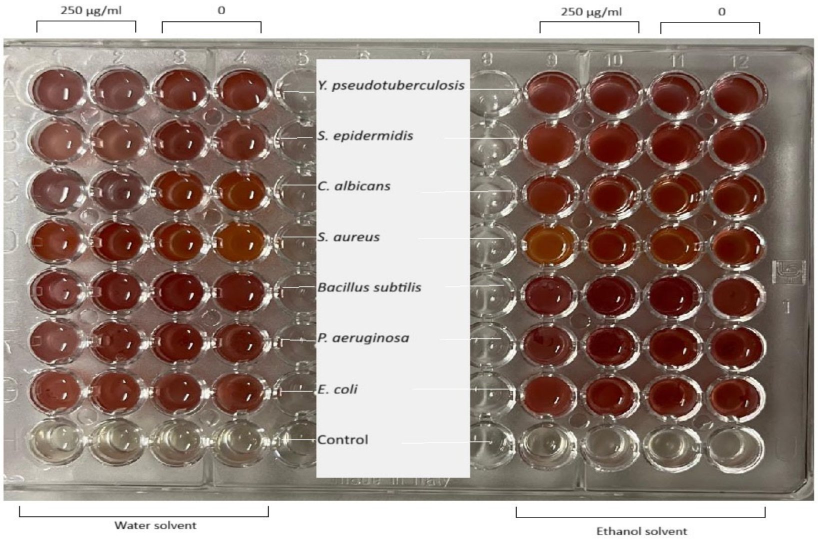

INT colorimetric assay for ethyl acetate (up), water, and ethanol (250 µg/ml) (down). The vitality of microorganisms did not decrease. There is no difference compared to the control without any solvent.

Table S2. Antimicrobial results of ethyl acetate, water and ethanol solvent.

	Ethyl acetate (µg/ml)		Water (µg/ml)		Ethanol (µg/ml)	
**Microorganisms**	**250**	**0**	**250**	**0**	**250**	**0**
B. subtilis	N.E.	N.E.	N.E.	N.E.	N.E.	N.E.
S.epidermidis	N.E.	N.E.	N.E.	N.E.	N.E.	N.E.
S. aureus	N.E.	N.E.	N.E.	N.E.	N.E.	N.E.
K.pnemonia	N.E.	N.E.	N.E.	N.E.	N.E.	N.E.
Y. pseudotuberculosis	N.E.	N.E.	N.E.	N.E.	N.E.	N.E.
E. coli	N.E.	N.E.	N.E.	N.E.	N.E.	N.E.
P.aeroginosa	N.E.	N.E.	N.E.	N.E.	N.E.	N.E.
C. albicans	N.E.	N.E.	N.E.	N.E.	N.E.	N.E.

MIC values of some extracts could not be determined and were expressed as 'No Effect' in Table.
